# Defining a minimal cell: essentiality of small ORFs and ncRNAs in a genome-reduced bacterium

**DOI:** 10.15252/msb.20145558

**Published:** 2015-01-21

**Authors:** Maria Lluch-Senar, Javier Delgado, Wei-Hua Chen, Verónica Lloréns-Rico, Francis J O'Reilly, Judith AH Wodke, E Besray Unal, Eva Yus, Sira Martínez, Robert J Nichols, Tony Ferrar, Ana Vivancos, Arne Schmeisky, Jörg Stülke, Vera van Noort, Anne-Claude Gavin, Peer Bork, Luis Serrano

**Affiliations:** 1 EMBL/CRG Systems Biology Research Unit, Centre for Genomic Regulation (CRG)Barcelona, Spain; 2 Universitat Pompeu Fabra (UPF)Barcelona, Spain; 3 European Molecular Biology LaboratoryHeidelberg, Germany; 4 Theoretical Biophysics, Humboldt-Universität zu BerlinBerlin, Germany; 5 Department of Genetics, Stanford UniversityStanford, CA, USA; 6 Vall d'Hebron Institute of Oncology (VHIO)Barcelona, Spain; 7 Department of General Microbiology, Institute for Microbiology and GeneticsGöttingen, Germany; 8 Centre of Microbial and Plant Genetics, KU LeuvenLeuven, Belgium; 9 Max-Delbrück-Centre (MDC) for Molecular MedicineBerlin, Germany; 10 Institució Catalana de Recerca i Estudis Avançats (ICREA)Barcelona, Spain

**Keywords:** minimal genome, non-coding RNAs, small proteins

## Abstract

Identifying all essential genomic components is critical for the assembly of minimal artificial life. In the genome-reduced bacterium *Mycoplasma pneumoniae*, we found that small ORFs (smORFs; < 100 residues), accounting for 10% of all ORFs, are the most frequently essential genomic components (53%), followed by conventional ORFs (49%). Essentiality of smORFs may be explained by their function as members of protein and/or DNA/RNA complexes. In larger proteins, essentiality applied to individual domains and not entire proteins, a notion we could confirm by expression of truncated domains. The fraction of essential non-coding RNAs (ncRNAs) non-overlapping with essential genes is 5% higher than of non-transcribed regions (0.9%), pointing to the important functions of the former. We found that the minimal essential genome is comprised of 33% (269,410 bp) of the *M. pneumoniae* genome. Our data highlight an unexpected hidden layer of smORFs with essential functions, as well as non-coding regions, thus changing the focus when aiming to define the minimal essential genome.

## Introduction

Defining the minimal genome that is required for sustaining life is currently one of the major challenges in biology. The essential genome of an organism, aside from protein-coding regions (ORFs), comprises regulatory (5′-UTRs and non-coding RNAs (ncRNAs)) and structural elements (Gil *et al*, 2004; Christen *et al*, 2011). Most of the previous essentiality studies (Glass *et al*, 2006; Lluch-Senar *et al*, 2007; French *et al*, 2008) made use of the conventional genome annotations which are biased against small proteins (smORFs; < 100 aa) (Samayoa *et al*, 2011) and regulatory elements such as ncRNAs. However, an accurate essentiality study is limited by the completeness of the genome annotation. Therefore, *M. pneumoniae* is an ideal organism due to its reduced genome size (816 kb) (Guell *et al*, 2009; Kuhner *et al*, 2009; Yus *et al*, 2009, 2012; Schmidl *et al*, 2010; Maier *et al*, 2011; van Noort *et al*, 2012; Lluch-Senar *et al*, 2013) and its detailed genome annotation based on experimental data. The current annotation of the *M. pneumoniae* genome contains 694 ORFs (32 of which are smORFs), 311 ncRNAs and 43 conventional RNAs (tRNAs, rRNAs, etc.) (Supplementary Table S2); all genes are well supported by transcriptome data, or in combination with proteome data [Supplementary Materials and Methods or http://mycoplasma.crg.eu/ for details (Wodke *et al*, 2014)]. This fine annotation of *M. pneumoniae* has been facilitated by the vast “-omics” datasets collected over the years (Guell *et al*, 2009; Maier *et al*, 2011; Yus *et al*, 2012), providing a better chance to gain a biased view on all putative essential elements in a minimal cell.

## Results and Discussion

**Figure 1:**
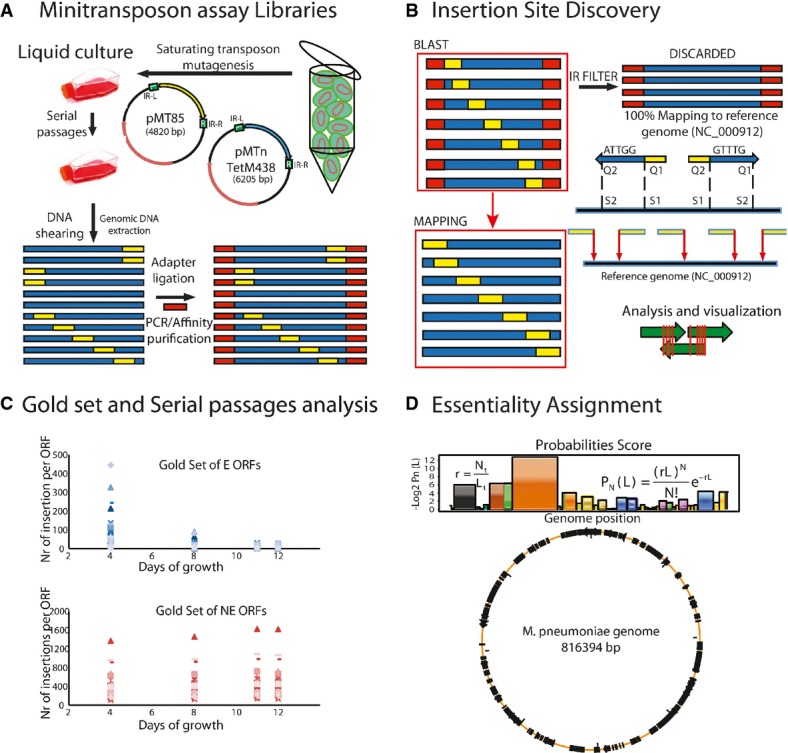
Transposon analysis

To determine the essentiality map, we used two mini-transposon mutant libraries (differing in the antibiotic resistance) of *M. pneumoniae* (Fig1A and B, Materials and Methods) and high-throughput insertion tracking by deep sequencing (HITS) (Wong *et al*, 2011) of cells at different days and serial passages (Fig1C). We analyzed day 12 sample since the number of insertions for the essential ORF gold set is close to zero, while this number for non-essential genes remains approximately constant (Fig1C) (Supplementary Table S1). We found a small insertion bias against G/C-rich quadruplet base sequences, but this does not affect the essentiality of smORFs since they have a similar composition as ORFs (Supplementary Materials and Methods). Based on the number of reads per insertion in the essential and non-essential gold sets, we define two thresholds to decide whether an insertion was annotated or not (a relaxed one with seven reads per insertion, and a stringent one with 41 reads) (Supplementary Materials and Methods). In the following, unless specified, we used the stringent value.

Mini-transposon assay libraries. Map of pMT85 and pMTnTetM438 vectors and schematic representation of the procedure to obtain the mini-transposon libraries. Cells were grown in liquid culture (two serial passages) after transformation. Then, genomic DNAs were isolated and libraries were prepared for sequencing by HITS. Blue indicates regions of the *M. pneumoniae* genome, yellow represents transposon insertion sites, and red adaptor sequences.

Insertion site discovery. Reads were first filtered by inverted repeat sequences and then mapped to the reference genome of *M. pneumoniae*. Insertion sites were defined by BLAST. Green arrows indicate ORFs and red lines transposon insertion sites.

Determination of the optimal number of cell passages and culture days required for analyzing essentiality. Blue dots, in the upper graph, indicate the number of insertions for each gene in the essential gold set. Pink dots, in the lower graph, indicate the number of insertions for genes in the non-essential gold set. Growth of 11 to 12 days allows the best separation in terms of number of insertions for the essential and non-essential gold sets.

Essentiality assignment. Upper panel shows the equations used to calculate the essentiality probabilities *P*_N_(L) with an example of a genome region (Supplementary Materials and Methods for the description of the formula). Lower circle represents essential regions in *M. pneumoniae* genome (in black); orange line represents non-essential regions.

The resulting integrated essentiality map (Supplementary File S1) after 12 days of growth consists of 69,994 unique mini-transposon insertions with a resolution of ∽4 bp for non-essential genes. Based on the analysis of the gold sets of essential and non-essential ORFs (Supplementary Table S1), we developed an essentiality probability criterion (Supplementary Materials and Methods; Fig1D) (Christen *et al*, 2011). Using this criterion, the 694 annotated ORFs were assigned to three distinct categories: essential (E; 342 ORFs), non-essential (NE; 259 ORFs) and fitness (F; 93 ORFs) (Supplementary Table S2) (Christen *et al*, 2011). The robustness of the classification was validated by the ability to isolate 92% of randomly selected F (12 genes) and NE (24 genes) clones, and the lack of success for 90% of E ORFs (28 out of 31, Supplementary Table S2). The 3 isolated E clones come out as fitness with the relaxed seven reads per insertion threshold, suggesting that they are severely affected in their growth. Moreover, when comparing with the predicted set of the minimal protein machinery in mollicutes, including 129 genes (Grosjean *et al*, 2014), we find 92% of them essential and 7% fitness. The dependency on the number of reads per insertion cutoff on fitness genes indicates that some of them could be incorrectly classified as essential when it is too strict. On the other hand, relaxing this cutoff results in some gold set essential genes being classified as fitness. This illustrates the limitation of transposon essentiality studies using deep sequencing for fitness genes.

**Figure 2:**
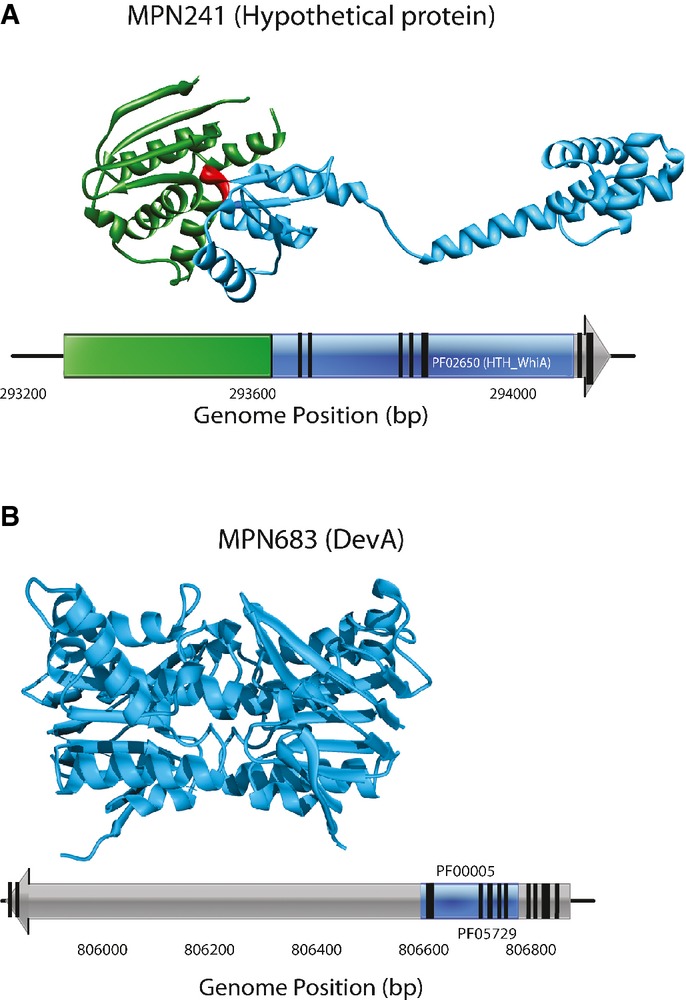
Essentiality at the protein domain level The crystal structures of the respective protein (homology modeled). Essential domains are highlighted in green and non-essential ones in blue. Regions with no Pfam domain assigned are shown in gray. The dashed lines indicate the start and the end of the aa sequence of each protein.

Notably, we found that the insertions were not evenly distributed along the entire ORFs as previously observed in *Caulobacter crescentus* (Christen *et al*, 2011). In this respect, it is important to note that our mini-transposon has an internal promoter that could allow expression of downstream genes or domains if there is a start codon for translation. This hints at the existence of individual domains that mediate the interactions within sub-complexes. Indeed, we found that multi-domain proteins involved in protein complexes are frequently more essential than proteins with a single domain and they are involved in important cellular processes such as transcription and DNA replication (Supplementary Fig S1). Analyzing the essentiality of individual protein domains revealed that in 81 multi-domain proteins, the essentiality status of individual structural domains differs (Fig2, Supplementary Table S2, Supplementary Materials and Methods). Furthermore, cloning and expression of some of these structural domains (C-terminus of MPN241, Fig2A and N-terminus of MPN683, Fig2B) showed autonomous folding since they can be expressed in a soluble manner (Supplementary Fig S2). These results indicate that identification of a transposon insertion as criterion for protein essentiality should be revised and domain essentiality analysis should be routinely applied instead.

MPN241, based on the crystal structure of the ortholog ORF in *Thermotoga maritima* (3HYI) (Kaiser *et al*, 2009).

MPN683, the structure of the ortholog protein in *Methanocaldococcus jannaschii* is shown (1L2T) (Smith *et al*, 2002).

**Figure 3:**
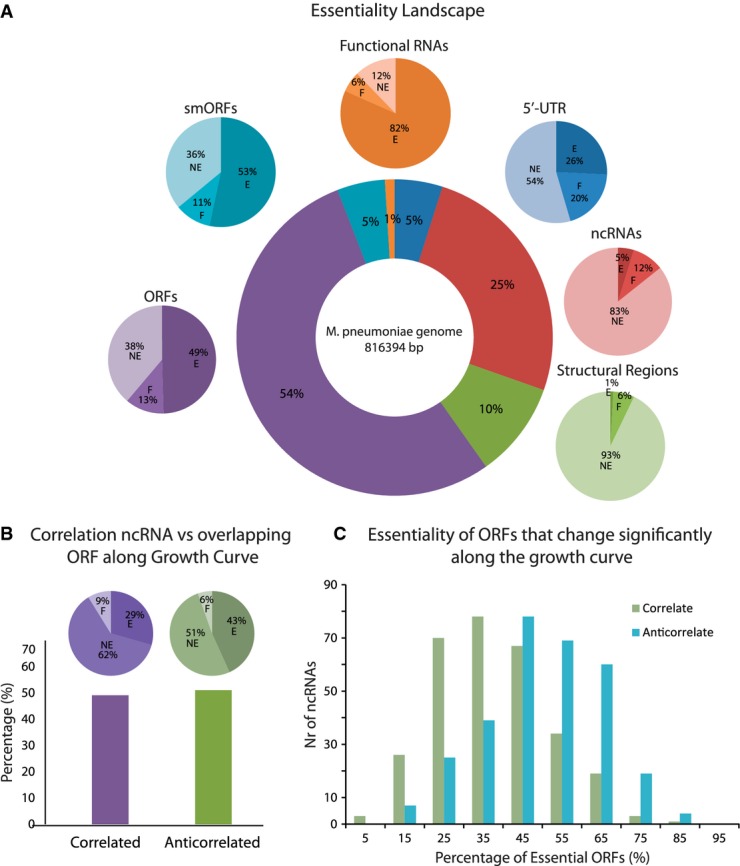
Essentiality genomic landscape and ncRNAs

Within non-transcriptionally active sequences of the *M. pneumoniae* genome, we detected 0.9% of essential intergenic regions (> 100 bp), which may function as structural elements including the origin of replication (oriC) (Fig3A, Supplementary Fig S3, Supplementary Table S3). In addition, we found that the percentages of essential transcriptionally active 5′-UTRs and ncRNAs (intergenic and overlapping with non-essential genes; for those overlapping with essential genes, no essentiality could be assigned) are 26 and 5%, respectively, and for conventional RNAs 82% (Fig3, Supplementary Table S2, Supplementary Materials and Methods). Strikingly, a large number of the ncRNAs (∽95%) overlap with coding genes on the opposite strand, which suggests that they have regulatory roles in gene expression. To gain insight into their functionality, we studied the correlation of expression of ncRNAs with their overlapping ORFs along 10 different time points of the growth curve by RNAseq. Interestingly, ncRNAs that anti-correlate with the overlapping ORF have higher essentiality coefficients than those that correlate (Fig3B, Supplementary Table S4). More importantly, the percentage of essential anti-correlated ORFs is higher than that of correlated ones (Fig3C; means of percentages: 50% versus 37%, respectively; *P* = 5.63e-10 applying Welch's two sample *t*-test), suggesting that essential ORFs are down-regulated by ncRNAs.

Essentiality landscape of the *M. pneumoniae* genome. Circle in the center represents the percentages of the different genomic elements with respect to the chromosome length. The linked pie charts show the essentiality of each element (represented in the same color; E is essential, NE is non-essential, and F is fitness).

The histogram represents the percentage of antisense ncRNAs that anti-correlate and correlate with their overlapping ORF along different time points of the growth curve (Supplementary Fig S7). The significant interactions (CLR score > 2.5) were determined for all pairs. Pearson correlations were used to determine whether the significant interaction is correlation or anti-correlation. The pie charts indicate the essentiality of the correlated and anti-correlated ncRNAs.

The graph indicates the distributions of the percentages of essentiality of all ORFs whose expression is correlating and anti-correlating with a given ncRNA.

**Figure 4:**
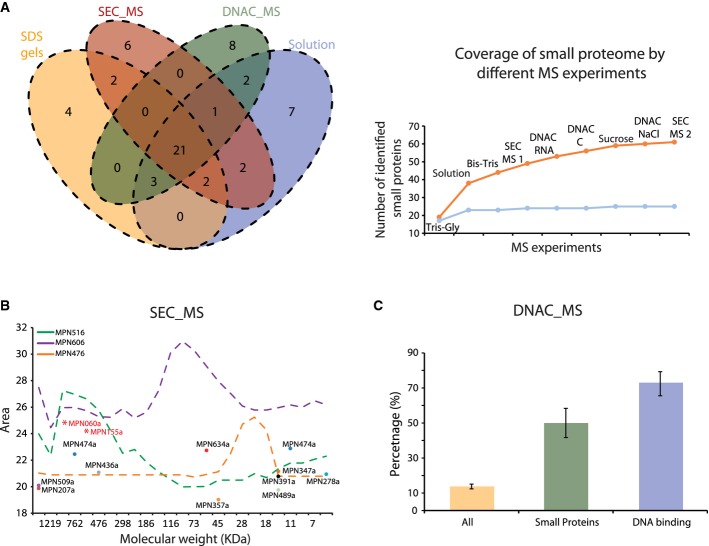
Functionality of smORFs

It is possible that some ncRNAs encode for smORFs similar to some long ncRNAs in eukaryotes (Cohen, 2014). In fact, smORFs have been found in bacteria associated with a diverse set of cellular functions (Hobbs *et al*, 2011; Samayoa *et al*, 2011). To investigate this, we translated all ncRNAs in the three reading frames and identified the putative ORFs by sequence searches and by combining mass spectroscopy (MS) with protein fractionation methodologies. Sequence conservation analysis with other bacterial species predicted eleven possible smORFs (Supplementary Fig S3, Supplementary Table S5, marked with α), of which four were identified by MS. Interesting examples are as follows: MPN391a, a cysteine-rich peptide predicted to be involved in peroxide resistance (Zimmerman & Herrmann, 2005), MPN347a, as part of an anti-toxin pair (Supplementary Fig S4) (Liu *et al*, 2008), and MPN155a that is homologous to a putative RNA-binding protein, YlxR, (Osipiuk *et al*, 2001) and is found in the same operon (Supplementary Fig S4). Interestingly, each fractionation methodology revealed new smORFs (Fig4A) extending the number from 32 annotated smORFs (25 detected proteins, mostly ribosomal, 56%) to a total of 67 smORFs (∽9% of the total ORFs). Additional fractionation experiments did not further increase the number of smORFs, suggesting that we are close to defining the complete *M. pneumoniae* small proteome (under the experimental limitation of identifiable peptides by MS for smORFs, Fig4A). As observed for the conventional ORFs, smORFs are often highly transcribed and essential (53%) (Supplementary Table S5, Fig3A).

Venn diagram showing the number of smORF proteins identified by MS in different experiments. SDS gels: smORFs identified in SDS gels (*n* = 4); solution: smORFs identified from total protein extracts (*n* = 4); SEC_MS: smORFs identified in size-exclusion chromatography (*n* = 2); DNAC_MS: smORFs identified in DNA–cellulose columns (*n* = 3). The graph represents the increase in the number of detected new smORFs (in orange) and known smORFs (in blue) by using different MS approaches. The names of MS approaches are as follows: Tris-Gly (*n* = 2) and Bis-Tis (*n* = 2) indicate smORFs identified by different SDS gels; solution: smORFs identified from total protein extracts (*n* = 4); SEC_MS1: smORFs identified in the first experiment of size-exclusion chromatography (*n* = 1) and SEC_MS2: smORFs identified in the second experiment of size-exclusion chromatography (*n* = 1); DNAC_RNA, DNAC_C and DNAC_NaCl: smORFs identified in DNA–cellulose columns, eluted by using RNA, chromatin or NaCl, respectively (*n* = 3); and sucrose indicates the number of smORFs identified by sucrose cushion.

The graph represents the size-exclusion chromatography elution profiles of different proteins: MPN516 (rpoB, subunit of the RNA polymerase complex), MPN606 (enolase, 50 kDa) and MPN476 (cytidylate kinase, 24 kDa). The main elution peak for the identified smORFs is indicated by dots. MPN155a and MPN060a were found after overexpression as a TAP fusion.

The histogram represents the percentages of proteins in each category that have been identified to interact with DNA in affinity chromatography after receiving operating characteristic curve (ROC) analysis (Supplementary Materials and Methods). “All proteins” represent the percentage of all proteins excluding known DNA- and RNA-binding proteins (Supplementary Materials and Methods). “DNA binding” proteins show the percentage of known poly-nucleotide directly binding proteins (Supplementary Materials and Methods). Black bars indicate the standard deviation after considering replicates from three different experiments.

In order to get insight into the reasons behind the high essentiality of the smORFs, we first investigated whether they are part of large protein complexes as previously suggested for some smORFs (Gassel *et al*, 1999). By size-exclusion chromatography coupled to MS (SEC-MS), we found that the vast majority (31 out of 34; 11 new) of the detectable smORFs eluted in fractions of significantly higher molecular weight than expected from the size of the individual proteins. This indicates that smORFs are frequently associated within larger protein complexes (Fig4B, Supplementary Table S6) and probably this is the case for the majority of the smORF. For example, overexpressing two smORFs, MPN060a and MPN155a, not detected in the original SEC-MS experiments, we find them eluting in high molecular weight fractions (Fig4B, Supplementary Fig S3B). Second, we used DNA–cellulose (DNAC) affinity chromatography coupled to MS to analyze DNA- or RNA-binding properties (Mai *et al*, 1998). We found that out of 35 smORFs identified in this experiment (14 previously unknown, Fig4A), 42% of new smORFs (including the putative RNA-binding protein MPN155a, YlxR) bind to DNA/RNA, compared to 15% of the conventional ORFs (excluding well-known DNA and RNA directly binding proteins) in *M. pneumoniae* (Fig4C, Supplementary Materials and Methods).

Understanding the minimal set of essential genetic elements is important for several applications, ranging from synthetic biology approaches to drug targets identification in pathogenic bacteria (Gallagher *et al*, 2007). Based on our analysis, we conclude that essentiality should be considered at a protein domain resolution and that smORFs as well as regulatory elements (5′-UTRs and ncRNAs) are frequently essential genomic elements, considerably increasing the repertoire of building blocks that need to be considered for a minimal genome. Furthermore, we revealed a previously unknown layer of essentiality composed of smORFs that are likely to play important roles in protein complex functionality and DNA transcriptional regulation. Thus, it is crucial to more carefully consider smORFs in genome annotations as they can comprise 9% of the genome ORFs.

## Materials and Methods

The mini-transposon mutant libraries of *M. pneumoniae* were obtained after transforming with pMT85 and pMTnTetM438 vectors and doing serial passages (Supplementary Materials and Methods) (Pich *et al*, 2006). Genomic DNAs were collected using the Illustrabacteria genomic kit (GE) and sequenced with the HITS approach (Fig1A and B) using standard Illumina paired-end sequencing. Raw reads were filtered by inverted repeats (IR) and then mapped to the *M. pneumoniae* reference genome (NC_000912, NCBI) using BLASTs (Supplementary Table S7).

Two gold standard sets were manually assembled; one contained 37 protein-coding genes that are known to be essential, and the other contained 29 NE ORFs (Supplementary Table S1). The two datasets were evaluated using our mini-transposon library, and then, a scoring system was developed that consisted of two parameters, *P*_E_, the probability for a genomic region of being essential, and *P*_NE_, the probability of being non-essential rounded to two decimals (Supplementary Table S2). This analysis revealed three distinct groups of genes with 99% confidence (Supplementary Table S2): those that are essential (E; *P*_E_ > 0 and *P*_NE_ = 0), those that are non-essential (NE; *P*_E_ = 0 and *P*_NE_ > 0) and a third group with an intermediate essentiality score that we define as fitness (F; *P*_E_ > 0; *P*_NE_ > 0 or *P*_E_ = 0; *P*_NE_ = 0). The fitness category includes those genes that essentiality could depend on condition and transposon insertions and despite having an impact on growth, they do not affect cell viability.

To study whether the protein products of smORFs could be involved in protein complexes, ten smORFs were selected and cloned into vector pMT85-clpB-TAPtag SfiI/NotI (Kuhner *et al*, 2009). After transforming *M. pneumoniae*, the protein complexes were studied by molecular weight exclusion chromatography coupled to Western blot. Fractions from molecular weight exclusion chromatography were trypsin-digested and then subjected to MS (Supplementary Materials and Methods). DNA/RNA-binding proteins were identified by DNA–cellulose (DNAC) affinity chromatography coupled to MS (Supplementary Materials and Methods).

### Data availability

The raw data of transposon libraries and RNAseq have been submitted to the ArrayExpress database (http://www.ebi.ac.uk/arrayexpress) and assigned the identifier E-MTAB-3075 and E-MTAB-3076, respectively. Additionally, genome re-annotation and MS data used for identification of smORFs have been submitted to ProteomeXchange via the PRIDE database (http://www.ebi.ac.uk/pride) and assigned the identifier PXD001611.

## Supporting Information

Supplementary Information

Review Process File
