# Supplementary figures and images for: Defining a minimal cell: essentiality of small ORFs and ncRNAs in a genome-reduced bacterium
Source: Mol Syst Biol. 2015 Jan 21;11(1):780. doi: 10.15252/msb.20145558 (PMC4332154; doi:10.15252/msb.20145558)

Figure S1

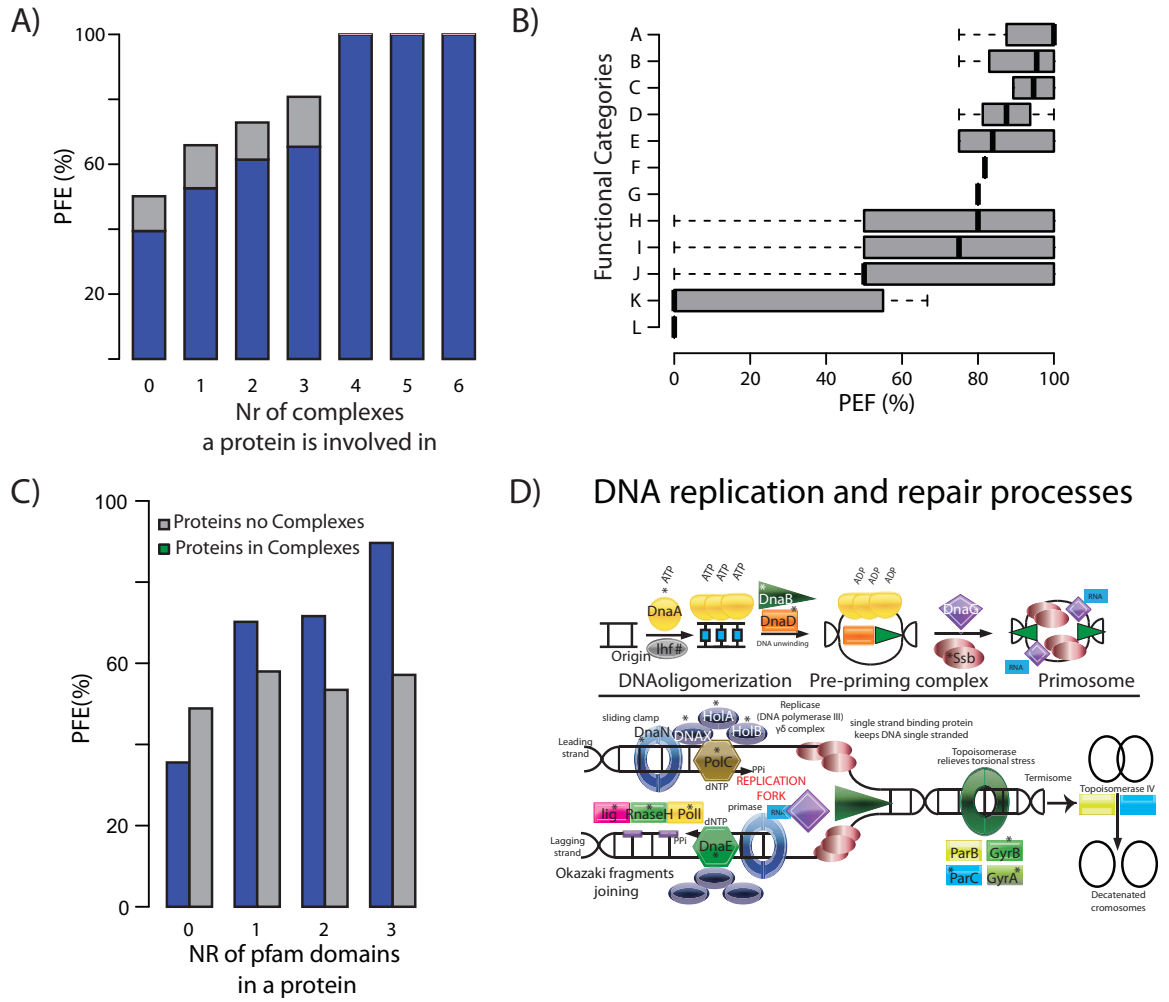

Supplement: Supplementary file 1 [file msb0011-0780-sd1.pdf]

Figure S2

A)

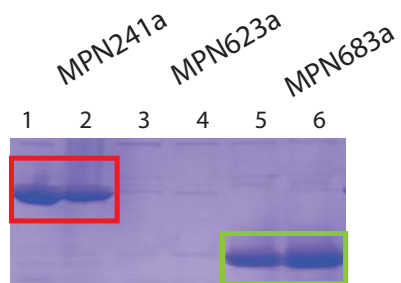

B)

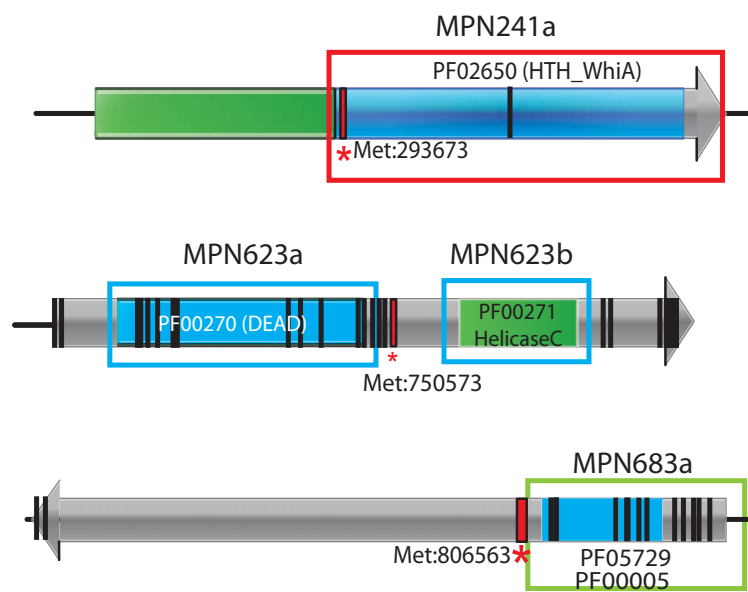

Supplement: Supplementary file 2 [file msb0011-0780-sd2.pdf]

Figure S3

A)

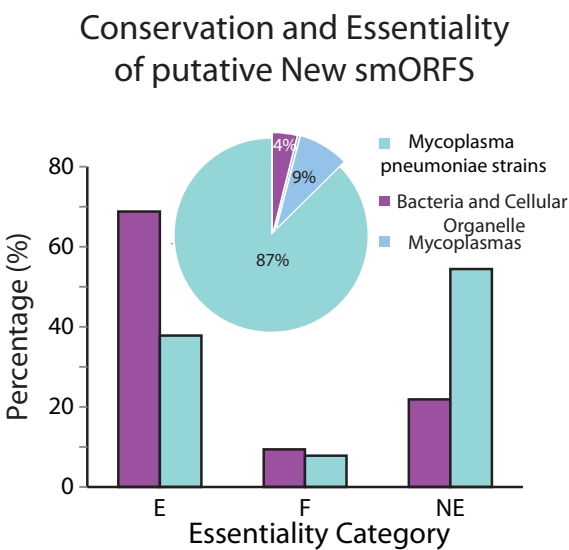

B)

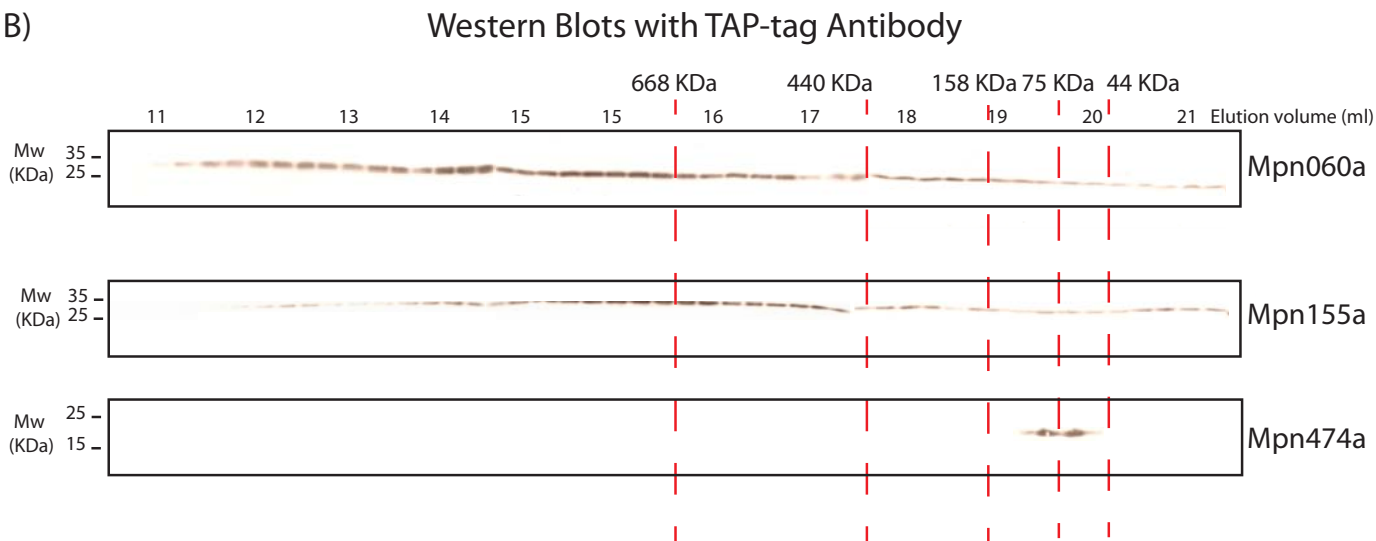

Supplement: Supplementary file 3 [file msb0011-0780-sd3.pdf]

Figure S4

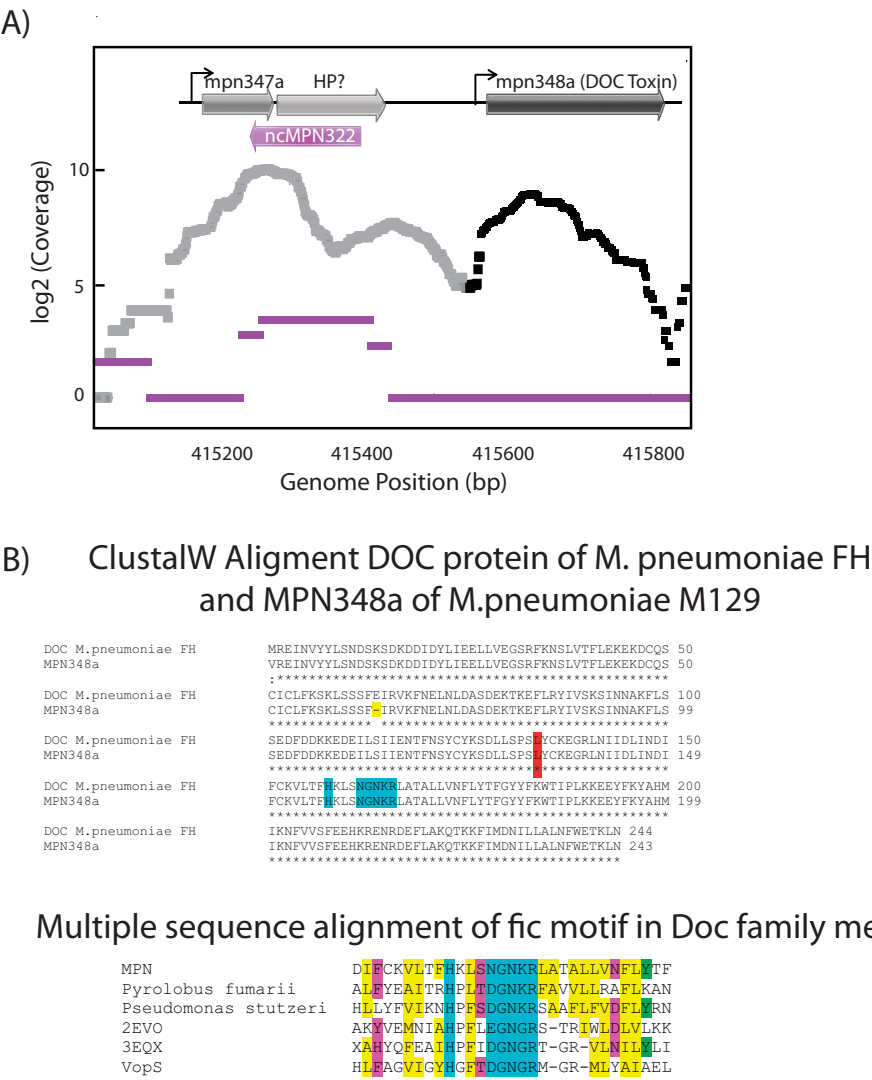

C) Conservation of genomic context mpn155a

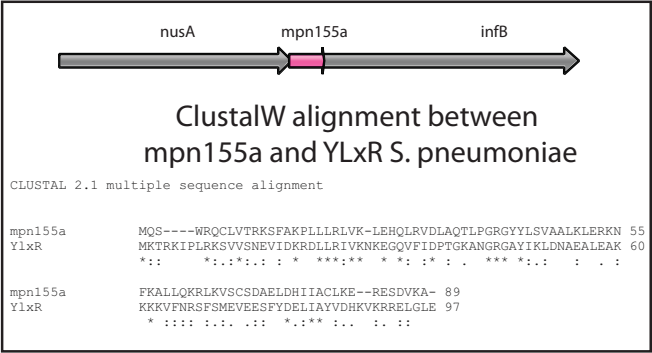

Supplement: Supplementary file 4 [file msb0011-0780-sd4.pdf]

Figure S5

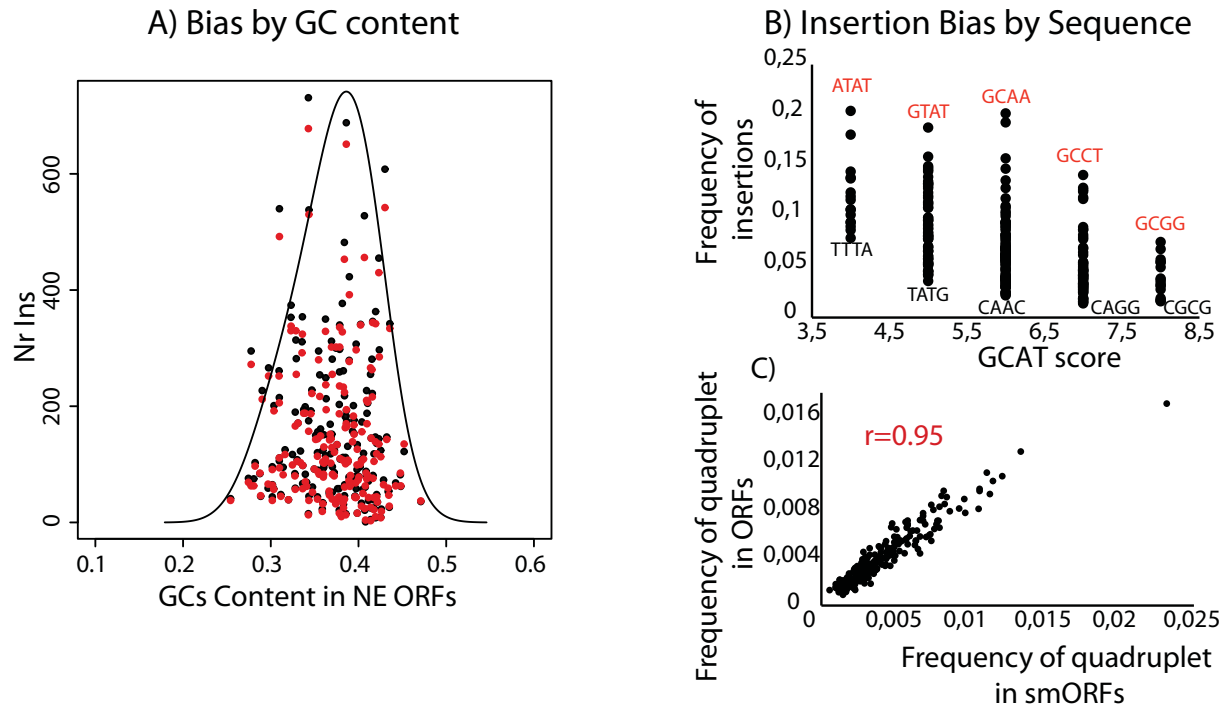

Supplement: Supplementary file 5 [file msb0011-0780-sd5.pdf]

Figure S7

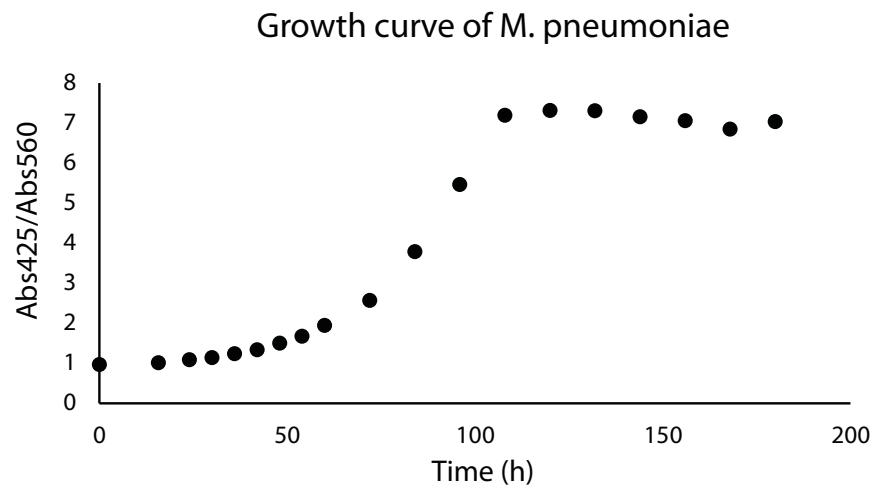

Supplement: Supplementary file 7 [file msb0011-0780-sd7.pdf]

Figure S8

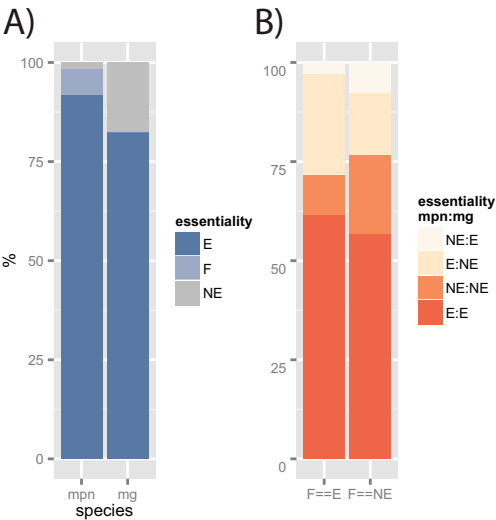

Supplement: Supplementary file 8 [file msb0011-0780-sd8.pdf]
