# Supplementary material for: Defining a minimal cell: essentiality of small ORFs and ncRNAs in a genome-reduced bacterium
Source: Mol Syst Biol. 2015 Jan 21;11(1):780. doi: 10.15252/msb.20145558 (PMC4332154; doi:10.15252/msb.20145558)
Supplement: Supplementary file 6 [file msb0011-0780-sd6.pdf]

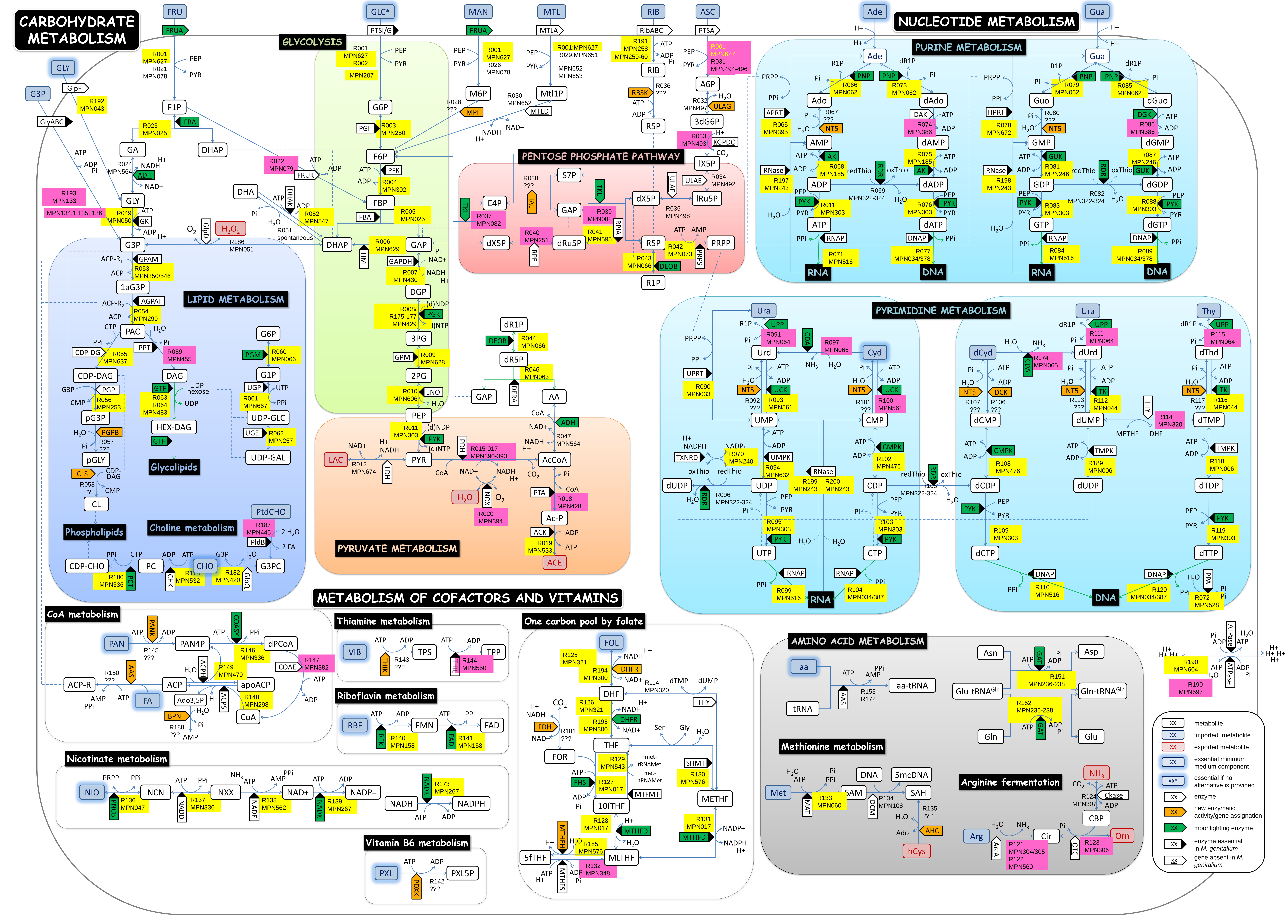
CARBOHYDRATE METABOLISM
GLYCOLYSIS
PENTOSE PHOSPHATE PATHWAY
LIPID METABOLISM
Phospholipids
Choline metabolism
Glycolipids
PYRUVATE METABOLISM
NUCLEOTIDE METABOLISM
PURINE METABOLISM
PYRIMIDINE METABOLISM
METABOLISM OF COFACTORS AND VITAMINS
CoA metabolism
Thiamine metabolism
Riboflavin metabolism
Nicotinate metabolism
Vitamin B6 metabolism
One carbon pool by folate
AMINO ACID METABOLISM
Methionine metabolism
Arginine fermentation
metabolite
imported metabolite
exported metabolite
essential minimum medium component
essential if no alternative is provided
enzyme
new enzymatic activity/gene assignation
moonlighting enzyme
enzyme essential in M. genitalium
gene absent in M. genitalium
