# Supplementary material for: Defining a minimal cell: essentiality of small ORFs and ncRNAs in a genome-reduced bacterium
Source: Mol Syst Biol. 2015 Jan 21;11(1):780. doi: 10.15252/msb.20145558 (PMC4332154; doi:10.15252/msb.20145558)
Supplement: Supplementary file 21 [file msb0011-0780-sd21.docx]

Supplementary Materials:

Supplementary Text:

Material and Methods

Supplementary Data

Figures S1-S9

Tables S1-S7

References (28-53)

**Supplementary Materials:**

**Title:** Defining a minimal cell: essentiality of small ORFs and ncRNAs in a genome-reduced bacterium

**Authors:** Maria Lluch-Senar^1,2†^, Javier Delgado^1,2†^, Wei-Hua Chen^3†^, Verónica Lloréns-Rico^1,2^ , Francis J. O’Reilly^3^, Judith A.H. Wodke^1,2,4^, E. Besray Unal^1,2^, Eva Yus^1,2^, Sira Martínez^1,2^, Robert J. Nichols^5^, Tony Ferrar^1,2^, Ana Vivancos^6^, Arne Schmeisky^7^, Jörg Stülke^7^, Vera van Noort^8^, Anne-Claude Gavin^3^, Peer Bork^3,9^, Luis Serrano^1,2,10^*

**Affiliations:**

^1^ EMBL/CRG Systems Biology Research Unit, Centre for Genomic Regulation (CRG), Dr. Aiguader 88, 08003 Barcelona, Spain

^2^ Universitat Pompeu Fabra (UPF), Dr. Aiguader 88, 08003 Barcelona, Spain.

^3^ European Molecular Biology Laboratory, Meyerhofstrasse 1, 69117 Heidelberg, Germany.

^4^ Theoretical Biophysics, Humboldt-Universität zu Berlin, Invalidenstr 42, 10115 Berlin, Germany

^5^ Department of Genetics, Stanford University, 300 Pasteur Drive, M-344, Stanford, CA 94305-5120

^6^ Vall d’Hebron Institute of Oncology (VHIO), Pg Vall d’Hebron 119-129, 08035 Barcelona

^7^ Institute for Microbiology and Genetics, Dept. of General Microbiology Grisebachstr.8, 37077 Göttingen, Germany

^8^ Centre of Microbial and Plant Genetics, KU Leuven, Kasteelpark Arenberg 22, 3001 Leuven, Belgium.

^9^ Max-Delbrück-Centre (MDC) for Molecular Medicine, Robert-Rössle-Str. 10, 13092 Berlin, Germany

^10^ Institució Catalana de Recerca i Estudis Avançats (ICREA), Pg. Lluis Companys 23, 08010 Barcelona, Spain

† contributed equally to this work.* to whom correspondence should be addressed: luis.serrano@crg.es

**Contents**:

Supplementary Text………………………...…………………………………….…….……..12

Supplementary Figures Legends……………………………………………………….…..….28

Supplementary Table Legends………………………………………………………………...31

References……………………………………………………………………………………..36

SUPPLEMENTARY TEXT

MATERIAL AND METHODS

**Bacterial strains and culture conditions**

*Escherichia coli* strain TOP 10 was grown at 37ºC in 2YT broth or LB agar plates containing 75 µg ml-1 ampicillin. *M. pneumoniae* was grown in 75 cm^2^ tissue culture flasks with 50 mL of modified Hayflick medium at 37ºC as previously described ([Yus et al., 2012](#_ENREF_32)). To select transformants for TAP-tag experiments media was supplemented with 100ug ml^-1^ gentamicin.

**Library of *M. pneumoniae* mini-transposon mutants**

*M. pneumoniae* M129 strain was transformed with pMT85 and pMTnTetM438 vectors. Those mini-transposon vectors are both derived from Tn4001 and they have different resistance markers under different promoters ([Pich et al., 2006](#_ENREF_24)). They are used to avoid bias by vector type in the essentiality study. After transformation, cells were spread on 75 cm2 flasks with 25 ml of Hayflick medium during 4 days. Then, two consecutive passages of the cells (4 days each) were performed and DNA was extracted in each passage by using the Illustrabacteria genomic Kit (GE). Then genomic DNAs were sequenced by HITS approach (Figure 1A).

**HITS approach**

Genomic DNAs were sheared to 100 bp DNA fragments by using a Covaris S2 device. Paired-end Illumina libraries were created as described by Bentley et al. ([Bentley et al., 2008](#_ENREF_1)). After adapters ligation, generated fragments were size selected (between 200 and 400 bp). Enrichment of transposon/chromosomal junction regions was performed by PCR amplification with a 5´- desthiobiotinylated primer specific of the inverted repeat (IR) sequence of transposon and adapter specific PCR PE 2.0 primer. Thermocycler settings were as follows: 30 s, 98°C; 18 cycles of 10 s, 98°C, 30 s, 65°C, 30 s, 72°C; 5 min, 72°C. Fragments between 250 and 300 bp were gel purified and added to Dynabeads® Magnetic Beads (Invitrogen) to capture desthiobiotinylated templates containing transposon insertions. Beads were washed according to the manufacturer’s instructions, and the transposon containing fragments were eluted with 2 mM of biotin. Supernatants were recovered from beads and the resulting transposon libraries were PCR amplified and quantified on an Agilent Bioanalyzer chip (Agilent Technologies). Double-stranded templates were cluster amplified and sequenced on an Illumina HiSeq 2000. The raw data of transposon libraries have been submitted to the ArrayExpress database (http://www.ebi.ac.uk/arrayexpress) and assigned the identifier E-MTAB-3075.

**Analysis and Mapping of Illumina Sequencing Data**

Standard Illumina paired-end sequencing was used and cluster generation was performed with the Illumina cluster generation protocol. Raw reads were filtered according to IR sequences. Velculescu *et al.* demonstrated that tags as short as 13 bp are enough to match DNA sequences, thus we selected reads containing 13 bp of the IR adjacent to genomic DNA. Filtered reads were mapped to the *M. pneumoniae* reference genome (NC_000912, NCBI) using Blast (maximum e-value=0.01). However, in some cases the minitransposon insertion promotes the duplication of the flanking region then this uncertainty could be 7bp. Transposon insertion sites and number of reads corresponding to each insertion, are shown in Table S8. Assignments of reads in repeated regions of the genome can be misleading. After identifying all the repetitive sequences of the genome, essentiality of regions containing them was re-calculated and marked with an asterisk in the Table S2. The data from sequencing runs of pMT85 (Gm) and pMTnTetM438 (Tet) samples after cell passages is summarized in Table S7.

**Study of bias by GC content**

Since Tn3 insertions have been shown to have a markedly stronger preference for an AT-rich, 5-bp target sequence ([Kumar et al., 2004](#_ENREF_13)), we examined whether pMT85 and pMTnTetM438 have an insertion bias. We did not detect any significant A/T sequence insertion bias for 200 bases regions when analyzing the essentiality of ORFs, ncMPNs and intergenic regions (Figure S5A). To look for specific sequence biases we determined how many insertions were found for each possible quadruplet (256 base combinations) and normalized this number by the occurrence of each quadruplet in the genome. Then we calculated a GCAT score (A,T=1, GC=2) for each quadruplet and assigned the insertion number corrected by their genome frequency (Figure S5B). Interestingly, quadruplets with high AT content show more transposon insertions. In quadruplets with a higher G/C content we find that quadruplets starting by GC have higher insertion frequency than others. To ensure that this bias does not have an effect in our essentiality study for smORFs we determined the frequency of occurrence of the 256 quadruplets for smORFs and ORFs. As a control we did several random sets of ORFs. We find that the overall correlation of quadruplet frequencies between smORFs and conventional ORFs was very high (r=0.95) (Figure S5C), and similar to that found between random sets of ORFs. This shows that the transposon insertion bias is affecting equally both genomic categories (Figure S5C).

**Essentiality scores, gold standard sets**

Our essential gold standard set is composed of :RNA polymerase subunits, sigma 70 factor, tRNA synthases, DNA polymerase complex, ribosomal RNA and the central glycolytic enzymes needed for ATP production ([Yus et al., 2009](#_ENREF_33)) (Table S1). The non-essential gold standard set is composed of a group of *M. pneumoniae* genes not found in the closely related species *M. genitalium* (Table S1). For each of the two gold standard sets, we calculated the average density of insertions at different cell passages (Figure 1C). The high sensitivity of deep sequencing allows the occasional detection of disruptive insertions in essential genes probably because such mutants, whilst unable to grow and divide, are still present at very low frequency on the initial selection plate of the respective mutant pool ([Christen et al., 2011](#_ENREF_2)). Such insertions are not generated by PCR amplification during sample preparation since PCR on the original pools detected all sequenced insertions in randomly selected essential ORFs, non-essential ORFs and fitness ORFs (Table S2). Interestingly, whilst density of insertions remain essentially constant with passage and day of culture in non-essential gold set, the density in essential gold sets decreases, obtaining the minimal value at 2 passages (12 days; Figure 1C). Thus, genome essentiality was evaluated after 2 passages (12 days). The high sensitivity of HITS still allow the detection of insertions in essential genes either because of attachment to other clones or because although they cannot divide or growth still could be alive. However, when we look at the number of reads per insertion for the essential gold set we see that mean of these numbers (8.9 in pMT85 and 7.5 in pMTnTetM438) is much lower than that for non-essential genes (24.5 in pMT85 and 23 pMTnTetM438). To decide the best number of reads to set a threshold we did a ROC curve for the two independent experiments (the two antibiotic resistances, Gentamycin (Gm) and tetracycline (Tet)). We also looked at which threshold for the number of reads we found the higher ratio between density of insertions per base between essential and non-essential genes. Based on these two analyses we used two filters of 7 and 41 reads (Thr7 and Thr41, respectively). Number of insertions per ORF in Gm and Tet samples revealed that both experiments correlate (r=0,997) indicating that both samples can be considered as biological replicates for the essentiality study. Thus, we merge the insertions of both experiments obtaining a total number of unique insertions of 237.001 and 69.994 for Thr7 and Thr41, respectively. Due to the 2 bp and 4bp resolutions for Thr7 and Thr41 respectively, we were able to evaluate the essentiality of small genes (~60-70 bases), overlapping ORFs and the effect of neighboring operons or regulatory regions.

The first or last bases in a gene might be essential due to proximity to, or an overlap with, an essential gene in the same or opposing strand. Also it is well known that in some proteins the first or last amino acids of a protein are non-essential ([Glass et al., 2006](#_ENREF_8); [Hutchison et al., 1999](#_ENREF_10)). Thus, we studied change in essentiality after removing the first and last bases of every ORF corresponding to 10% of gene length.

The density of insertions (R) for each gold set after applying 7 and 41 thresholds was calculated and values were integrated in a Poisson distribution probability equation ([Osterman and Gerdes, 2008](#_ENREF_23)):

whereby L is the length of the region, R is the linear insertion density and N the insertion number for the region. For each of the two gold standard sets we calculated R (for essential genes, R_E_ = 0.0013/base, and non-essential R_NE_ = 0.613/base for Thr7; and R_E_ = 0.0015/base, assuming that by chance there could be 2 insertions per gene, and R_NE_ = 0.14/base for Thr41).

Then, for each of the 694 validated and re-annotated ORFs, 311 ncMPNs, non-transcribed regions, as well as 5´-UTRs longer than 70 bp, we determined the probability values to be essential, P_E_ and non-essential, P_NE_. This analysis revealed three distinct groups of genes with 99% confidence (Table S2): those that are essential (E; P_E_ >0 and P_NE_ =0), those that are non-essential (NE; P_E_ =0 and P_NE_ >0) and a third group with an intermediate essentiality score that we define as fitness (F; P_E_ >0; P_NE_ >0 or P_E_ =0; P_NE_ =0).

We estimated the minimal genome by considering the analysis of Thr41 since it is the most restrictive and allows the best separation of essential and non-essential regions, but we also show Thr7 since three gens that have disruption in genes considered to be E at Thr41 were isolated in our work and they are fitness at Thr7. This suggests that genes that have a strong fitness effect could be removed or lost from a transposon library upon serial passage or being too strict in the cutoff. Thus we place high confidence on genes that are E, F and NE in both analysis, and we have less confidence in genes that change category.

**Score validation**

The scoring function was validated using two approaches: *i)* confirmation of insertions by PCR amplification from genomic DNA of individual pools and posterior sequencing. This approach not only confirmed insertions but also discarded putative non-specific amplifications by PCRs in library preparation; and *ii)* isolation of transposon mutants from the pools to assess the viability of the different clones and to determine if the criteria are robust. The different tags assigned to the different samples allowed the original pool where the mutants were located to be determined, thus facilitating their location and posterior isolation. To isolate the different clones, mycoplasma cells were passed several times through a syringe and then spread onto Hayflick agar plates. Single colonies were picked and grown in 5ml of Hayflick media at 37ºC. The purity of the clones was assessed by PCR amplification of the ORFs. If the wild type copy was detected, the procedure was repeated until single and pure clones were obtained.

**Identification of putative modular proteins**

The probability values P_E_ and P_NE_ were calculated for every genome position considering a sliding window of 200 bp. Determination of Pfam and InterPro domains allowed us to identify the domain essentiality and to check if the essential regions of an ORF correspond to putative functional regions or active protein domains.

**Recombinant expression of domains of putative modular proteins**

Cloning of domains of MPN241, MPN623, MPN683 in pETM14 ccdB was done using Gibson assembly (Table S9; Supplementary Materials). Mutation of TGA (W in *M. pneumoniae* but Stop in *E. coli*) codons to TTG was required for protein expression of MPN683 and MPN623 in *E. coli*. The domain of MPN241 corresponds to the 196 aa of the C-terminal protein (33% of the protein); the C-terminal domain of MPN683 protein corresponds to 162 aa (48% of the protein); and two fragments of MPN623 corresponding to 194 aa from the N-ter (43% of the protein length) and 210 aa from the C-ter (47% of the protein length). Two different *E. coli* strains (BL21 (DE3) pLys and BL21 (DE3) pRARE) were used to over-express these domains and expression was induced with 1mM of IPTG at 37ºC and 16ºC for 2h and overnight, respectively. After inductions and cell lysis by sonication, soluble and insoluble fractions were separated by centrifugation 15’ at 18.000 g (JA20 rotor Beckman). Proteins were separated by using 4-12% Bis Tris pre-casted protein gels following the instructions of the manufacturer (Life Technologies).

**RNA extractions and sample preparations**

After growing *M. pneumoniae* during 1h, 2h, 6h, 8h, 10h, 12h, 24h, 36h, 48h and 72h at 37°C, cells were washed twice with PBS and lysated with 700 µl of Qiazol buffer. Then, samples were lysated with 700 µl of Qiazol buffer. RNA extractions were performed by using miRNeasy mini Kit (Qiagen) following the instructions of the manufacturer. Libraries for RNA-seq were prepared following directional RNA-seq library preparation and sequencing. Briefly, 1 µg of total RNA was fragmented to ~100-150 nt using NEB Next Magnesium RNA Fragmentation Module (ref. E6150S, NEB). Treatments with Antarctic phosphatase (ref. M0289S, NEB) and PNK (ref. M0201S, NEB) were performed in order to make the 5’ and 3’ ends of the RNA available for adapter ligation. Samples were further processed using the TruSeq small RNA Sample Prep Kit (ref. RS-200-0012, Illumina) according to the manufacturer's protocol. In summary, 3’ adapters and subsequently 5’ adapters were ligated to the RNA. cDNA was synthesized using reverse transcriptase (SuperScript II, ref. 18064-014, Invitrogen) and a specific primer (RNA RT Primer) complementary to the 3’ RNA adapter. cDNA was further amplified by PCR using indexed adapters supplied in the kit. Finally, size selection of the libraries pas performed using 6% Novex® TBE Gels (ref. EC6265BOX, Life Technologies). Fragments with insert sizes of 100 to 130 bp were cut from the gel, and cDNA was precipitated and eluted in 10 µl of elution buffer. Double-stranded templates were cluster amplified and sequenced on an Illumina HiSeq 2000. The raw data of RNAseq have been submitted to the ArrayExpress database (http://www.ebi.ac.uk/arrayexpress) and assigned the identifier E-MTAB-3076.

**Analysis of RNASeq Data**

The Illumina TrueSeq stranded library protocol was utilized to determine expression along the growth curve of *M. pneumoniae*. 10 time-points of the growth curve were studied: 1 hour (h), 2h, 6h, 8h, 10h, 12h, 24h, 36h, 48h, and 72h. For each experiment, both ends were treated as independent single-end reads in order to avoid wrong assignment of read-pairs. Filtered reads were mapped to the *M. pneumoniae* reference genome (NC_000912, NCBI) using Maq mapping software. We mapped the reads containing 50 bp and allowing for 1 mismatch. The counts per kb per million reads (CPKM) value was calculated for each ORF and ncRNA as follows:

$$CPKM=\frac{counts per gene}{({total counts}/{{10}^{6}) x ({gene length}/{{10}^{3})}}}$$

The CPKM vales were converted to log2 values. The growth curve of 10 time-points was used for the further step.

**Statistical analysis of correlations.**

The fold-changes of the 10 consecutive time-points of growth curve were used to determine the anti-correlation of each annotated region of *M. pneumoniae*. The Pearson correlation of each pair was calculated via an R script. In order to determine the significant (anti)correlations, context likelihood relatedness (CLR) was employed ([Faith et al., 2007](#_ENREF_5)). CLR is mainly used to determine the direct interactions between genes for gene regulatory network construction; it is based on mutual information of a given expression dataset. CLR determines the strength of interaction between 2 genes by comparing this interaction to background distribution of all interactions of those target genes. For the growth curve, exponential phase and late phase the CLR interaction matrix were formed with the minet package of R ([Meyer et al., 2008](#_ENREF_20)). The significant interactions (CLR score > 2.5) were determined for all pairs. The anti-sense ncRNA of ORFs were analyzed in detail. Since the CLR score is always a positive value, the pre-calculated Pearson correlations were used to determine if the significant interaction is correlation or anti-correlation.

**Identification of SmORFs**

1. **Glycine and Tricine SDS gels.**

*M. pneumoniae* strain M129 was grown in 75 cm2 flasks for 72h. Harvested cells were washed twice with PBS buffer and lysed by addition of standard SDS loading buffer (Life Technologies) in a final volume of 200 µl of PBS. Two Novex® NuPAGE® Bis-Tris and two Novex® Tris-Glycine gels  (Life Technologies) were used to separate the proteins from the total cell extract of two biological replicates (15 µl loaded in each lane). Protein extract from two biological replicates were loaded in two lanes respectively of the 4 different gels (Two glycine and two Tris). Sixteen different gel bands (each band comprising the two biological replicates of each gel) were cut covering all the lanes length. In total 32 samples derived from Novex® NuPAGE® Bis-Tris gels and 32 samples derived from Novex® Tris-Glycine gels were analyzed by MS.

1. **Total protein extract along growth curve**

*M. pneumoniae* strain M129 was grown for 6 h, and 96 h at 37°C cells. Then, the medium was removed and cells were washed twice with PBS. Total protein extract was obtained by breaking the cells with 200 µl of lysis buffer (4% SDS, 0.1M DTT and 0.1M Hepes). Then total protein extracts of two biological replicates for each time-point were analysed by MS (see section iii).

1. **Molecular weight exclusion chromatography (SEC-MS).**

*M. pneumoniae* strain M129 was grown for 96 h in two flasks of 300 cm^2^ flasks, cells were collected in 1ml of lysis buffer (PBS, 2 mM MgCl2, 10 % glycerol with 4 ug/ml of DNAse 15’ at RT). Then cells were passed three times by the cell disruptor and cell extracts were centrifuged during 10’ at 18.000g (Beckman JA20 rotor) and 4 ºC. 250 µl of the soluble fraction was injected onto a Superdex 200 10/300 GL (24 ml) column and fractions of 500 ul were collected. The column was calibrated by using the protein molecular weight standards; Aldolase (4mg/ml; 158 kDa; 12.5 ml), Ovoalbumin (4mg/ml, 44 kDa, 14.5 ml); Ribonuclease (3mg/ml; 13.7 kDa, 17ml) and Aprotinin (3mg/ml; 6.5 kDa; 18.5 ml). Fractions of molecular weight exclusion chromatography corresponding to elution volumes 7.5 ml to 25.5 ml (samples named B10 to E3), were analyzed by MS and Western Blot.

1. **DNA binding columns**

*Mycoplasma pneumoniae* cells were diluted 1:10 in Hayflick (Yus, Guell et al 2009) and grown for 3 days at 37ºC in a 300 cm2 flask. Cells were washed with ice-cold PBS, collected in 5 ml of lysis buffer (50 mM Tris·HCl, 1 M NaCl, 1 mM CaCl2, 1 mM EDTA, 0.1% Triton X-100,  1 mM DTT, pH 8), supplemented with protease inhibitor tablets (Roche). Cell extracts were centrifuged for 30 min at 100.000xg and 4 ºC (Beckman ultracentrifuge) and the soluble fraction was diluted 10 times with 50 mM Tris·HCl, 1 mM CaCl2, 1 mM EDTA, pH 8 (to dilute out salt and detergent). The protein was loaded onto a DNA-Cellulose column (2g, Sigma) run on an Äkta Xpress (GE Healthcare) in equilibration buffer (50 mM Tris·HCl, 0.1 M NaCl, 1 mM CaCl2, 1 mM EDTA, pH 8). After washing thoroughly with equilibration buffer, nucleic acid binding proteins were eluted with 1 ml 1 M NaCl in equilibration buffer (Cellulose DNA binding in Table S5), or 5mg/ml yeast ribonucleic acid in TE (Cellulose RNA in Table S5), and concentrated by TCA precipitation. A cellulose resin was used as a negative control. Also we assess DNA binding properties by ultracentrifugation employing a sucrose cushion as previously described (([Prasad and Dritschilo, 1992](#_ENREF_26)) Chromatin sucrose cushion in Table S5). Proteins were then analyzed by Mass Spectroscopy (Orbitrap Velos Pro, CRG facility).

**Proteomics analysis**

Each fraction (amounts ranging from 20 to 486 µg) was digested in solution with trypsin. Briefly, samples were dissolved in 6 M urea, reduced with dithiothreitol (10 mM, 37 ºC, 60 min), and alkylated with iodoacetamide (20 mM, 25ºC, 30 min). Samples were diluted 10-fold with 0.2 M NH4HCO3 before being digested at 37 ºC overnight with trypsin (ration protein:enzyme 10:1). Peptides generated in the digestion were desalted, evaporated to dryness and dissolved in 300 µl of 0.1%FA. An aliquot of 2.5µl of each fraction (amounts ranging from 0.17 to 4 µg) was run on an LTQ-Orbitrap Velos (Thermofisher) fitted with a nanospray source (Thermofisher) after a nanoLC separation in an EasyLC system (Proxeon). Peptides were separated in a reverse phase column, 75 μm x 150 mm (Nikkyo Technos Co., Ltd.) with a gradient of 5 to 35% ACN with 0.1% FA in 60 min at a flow of 0.3 mL/min. The Orbitrap Velos was operated in positive ion mode with nanospray voltage set at 2.2 kV and source temperature at 325 °C. The instrument was externally calibrated using Ultramark 1621 for the FT mass analyzer and the background polysiloxane ion signal at m/z 445.120025 was used as lock mass. The instrument was operated in data-dependent acquisition (DDA) mode and in all experiments full-MS scans were acquired over a mass range of m/z 350-2000 with detection in the Orbitrap mass analyzer at a resolution setting of 60,000. Fragment ion spectra produced via collision induced dissociation (CID) were acquired in the ion trap mass analyzer. In each cycle of DDA analysis, following each survey scan the top twenty most intense ions with multiple charged ions above a threshold ion count of 5000 were selected for fragmentation at a normalized collision energy of 35%. All data were acquired with Xcalibur 2.1 software. 20 µg of the total extract was also digested and desalted and 1 µg of the resulting peptides analyzed on an Orbitrap Velos Pro in the same conditions as the fractions but with a longer gradient (120 min). Protein identification and quantitation was performed by Proteome Discoverer software v.1.3 (ThermoFisher) using MASCOT v2.3.1 (Matrix Science) as search engine. Data were processed by a search against a database that contains all the putative *M. pneumoniae* proteins larger than 19 aa and including the most common contaminants (86933 sequences). Carbamidomethylation for cysteines, was set as fixed modification, and acetylation in protein N-terminal and oxidation of methionine were set as variable modifications. Peptide tolerance was 7 ppm in MS and 0.5 Da in MS/MS, and a maximum of three missed cleavages were allowed. Peptides were filtered based on the FDR (False Discovery rate) lower than 5%.

Proteins were quantified using the T3PQ approach ([Ghaemmaghami et al., 2003](#_ENREF_7)) with an in-house R script that considered the average of the three most intense unique peptides without missed cleavages.

**smORFs identification**

To annotate a smORF we required at least a single peptide with a unique mass identified in more than one experiment, or more than one unique peptide if the protein was identified in a single experiment. Only peptides corresponding to the ORFs for which an RNA transcript could be identified were considered.

**Homology searches for smORFs**

To identify putative ortholog sequences of annotated *M. pneumoniae* smORFs, we searched the protein sequences as queries against 1) annotated proteins 2) six-frame translated genomic sequences of completely bacterial genomes in NCBI. An e-value cutoff of 0.002 was used for the NCBI BLAST tool. A BLAST hit in the first database above the threshold indicates that the smORF could be part of a larger gene, while a hit only in the second database indicates misannotation in the corresponding species. We found that most cases belong to the 2nd scenario, likely due to smRFs being often discarded in standard bacterial genome annotation.

**Functional characterization of smORFs: Complex formation and DNA binding**

Ten smORFs were selected to be overexpressed with a TAP-tag as previously described ([Kuhner et al., 2009](#_ENREF_12)). The different ORFs were amplified by PCR and cloned by using Gibson Assembly Cloning Kit (New England Biolabs) into pMT85-clpB-taptag SfiI/NotI digested vector. The 3 successful TAP-tag-protein expressing cell lines (TAP-mpn060a, TAP-mpn155a, TAP-mpn474a) were grown in 4x 300 cm^2^ plates and the cells were harvested and lysed as previously described ([Kuhner et al., 2009](#_ENREF_12)). 100 µl of the lysate was separated on a Biosep-SEC-s4000 (Phenomenex), flow rate 250 µl.min^-1^ and 250 µl fractions were collected. 100 µl from these resulting fractions were separated by SDS-PAGE and Western blotted. The blots were probed using the peroxidase-anti-peroxidase (PAP) antibody (Sigma), which recognizes the protein A moiety of the TAP-tag.

To evaluate if one protein can interact with DNA we calculate the enrichment by calculating the ratio of the areas between the control sample and the different elution fractions of the Cellulose DNA and RNA binding experiments (two elution fractions per experiment, injected twice in MS; a total of four replicates per experiment). We defined a gold set comprising known DNA binding proteins (30 ORFs; RNA polymerase, topoisomerases, DNA methylases, Transcription factors…). For each of the chromatography fractions analyzed by MS we determined the area under the curve of the top three peptides (or less if only one or two were identified) for each protein. Then, we calculated the area ratio between elution fractions and the flow through fraction.

To define the threshold we calculate the ROC curves of the two experiments (elution with salt and RNA), considering as true positive set the DNA-binding proteins and as true negative set, the rest of proteins (discarding DNA-binding, RNA-binding and smORFs). The area under the curves (AUC) and the calculated thresholds are indicated in Table S10. Then, by applying these thresholds, we estimated the percentage of proteins in each category that are found in each experiment. In figure 4 the histogram represents the average of the percentages from the two different experiments and the error bars indicate the differences respect to these values. All proteins are all the ORFs of *M. pneumoniae* genome (excluding smORFs and known DNA/RNA binding proteins); DNA binding represent the gold set defined above; and smORFs corresponds to the percentage of the 12 identified smORFs.

# SUPPLEMENTARY DATA

**ORF functional analysis of essentiality**

As the ORFs are better understood than other genomic regions and comprise 88% of the genome, we analyzed them in more detail. As expected, the translation machinery is essential as observed by enrichment in the functional categories translation, ribosomal proteins, and ribosome biogenesis functions among E ORFs (Applying Fisher’s test: OddRatio: 2.2 and P-value: 2.6 x 10^-7^). As found in *E. coli* and *S. cerevisiae* proteins ([Ghaemmaghami et al., 2003](#_ENREF_7); [Taniguchi et al., 2010](#_ENREF_30)), E and F ORFs have higher levels of protein and mRNA than NE ORFs in the exponential phase (P-value 1.5 x 10^-11^ for RNA levels and 1.5 x 10^-7^ for protein levels, applying a two sided T-test). Enriched categories for NE ORFs are: cell envelope biogenesis, outer membrane (P-value: 9.3 x 10^-11^), cell motility and secretion (P-value: 7 x 10^-4^), unknown function (P-value: 0.005) and defense mechanisms (P-value: 0.02), expected to be dispensable for *M. pneumoniae* growth ([Hutchison et al., 1999](#_ENREF_10)). Finally, enriched categories for F are metabolic genes (P-value: 0.01) and genes involved in cell division and chromosome partitioning (P-value: 0.05).

The set of essential and fitness ORFs of *M. pneumoniae* has the following features:

1. The complete machinery involved in DNA replication consisting of 17 essential ORFs (Figure S1D). Interestingly, *mpn014* (coding for a protein that contains a domain of DNA primase) is non-essential (Table S2). However, *mpn353* coding for DnaG is essential, suggesting that this gene is the one that has primase activity. Regarding nucleotide excision and DNA repair, there are nine essential four non-essential and two fitness genes.
2. The complete transcriptional machinery is essential or fitness, including the subunits of RNA polymerase, Sigma70, auxiliary subunits (*mpn401* (GreA), *mpn030* (NusB), *mpn067* (NusG), *mpn154* (NusA), *mpn164 (*NusE) and *mpn020* (YabA)), and three transcription factors identified by sequence homology (*mpn239* (GntR), *mpn266* (Spx) and *mpn478* (YrbC, a putative transcription factor of the YebC/TetR family)).
3. The nearly complete translational system, comprising ORFs involved in translation, ribosomal structure, and biogenesis is essential (109 out 119). Interestingly, 8 ORFs involved in translation are fitness. Only *mpn548* (RNA pseudouridylate synthase) is non-essential.

After the comparative of the 122 ortholog genes of *M. pneumoniae* out 129 recently suggested to be the minimal translational apparatus of Mollicutes ; only two were non-essential in our experiment, the others were either essential or fitness (98%), as shown in Figure S8A. As a comparison, we also identified 120 genes in *M. genitalium* genes that are orthologs to Grosjean's list, and found that a much larger fraction (~18%) is NE (Table S11; ([Glass et al., 2006](#_ENREF_8))).

1. Most of the genes of the cell division (dcw) operon (*mpn314-317*) are fitness (*mpn314, mpn315* and *mpn317*). The *mpn314* encodes for MraZ described recently in *E. coli* as transcriptional regulator ([Eraso et al., 2014](#_ENREF_4)) and *mpn315* gene codes for MraW, a S-adenosyl-methyltransferase involved in the methylation of 16S rRNA ([Kimura and Suzuki, 2010](#_ENREF_11)). The *mpn316* and *mpn317* genes, coding for a FtsA-like protein and FtsZ, are non-essential and fitness, respectively. FtsZ forms the Z-ring and recruits the other proteins necessary for constriction and achieving cytokinesis. Although FtsZ is essential for cell division in most bacteria, it was also shown to be non-essential in *M. genitalium* when cells can glide ([Lluch-Senar et al., 2010](#_ENREF_19)).

A comparison of our essentiality study and a previous metabolic flux balance analysis of *M. pneumoniae* ([Wodke et al., 2013](#_ENREF_31)) (Figure S6) showed that 43 out of 44 ORFs (98%) predicted to be essential *in silico* are also essential *in vivo*, while the other ORF (*mpn348*) is fitness. As expected, all genes involved in glycolysis are essential, as well as 5 out of 10 genes involved in pyruvate metabolism. Interestingly, in the metabolism of cofactors and vitamins, as expected, obtaining tetrahydrofolate (THF) from folate is an essential reaction. In the metabolism of amino acids, 4 genes of arginine fermentation pathway are fitness whilst *mpn307* is non-essential, (Figure S6). Regarding nucleotide metabolism, there is more than one pathway involved in the production of dUDP and several genes involved in the different reactions are fitness or even non-essential (*mpn322* to *mpn324*). This result suggests that in the absence of one pathway the other can be used to obtain the same metabolite. Detailed comparison of transposon essentiality with that obtained from flux balance analysis shows 61% agreement ([Wodke et al., 2013](#_ENREF_31)).

Additionally, comparative across *M. pneumoniae* and *M. genitalium* of 444 pairs of one-to-one ortholog genes ([Powell et al., 2014](#_ENREF_25)), about ~75% showed consistent essentiality statuses, i.e. the two genes of a ortholog pair are either both essential or both non-essential in the two mycoplasmas (Figure S8B; Table S11).

**Essentiality in protein complexes**

Essentiality can also go beyond single gene products and often expand to entire molecular machines, or protein complexes, that are the actual functional units in a cell. Thus, it is expected that gene products involved in complexes are most likely to have similar essentiality statuses. We identified series of essential protein complexes, i.e. containing more than 80% E or F ORFs (Figure S1A). They are involved in a variety of essential functions, including post-translational modification, protein turnover, chaperone metabolism, DNA replication, recombination and repair, carbohydrate transport and metabolism, transcription and translation, ribosomal structure and biogenesis (Figure S1B, and 1D). Notably, proteins involved in several complexes have a higher tendency to be essential (Figure S1A), and tend to have more functional domains (according to Pfam database ([Punta et al., 2012](#_ENREF_27))).

***M. pneumoniae* specific protein extensions and structural modularity**

Two well-known phenomena in bacteria are i) the expression of two polypeptides from a single transcript, and ii) the possibility of specific functional extensions of proteins ([Letunic et al., 2012](#_ENREF_16); [Mulder et al., 2002](#_ENREF_21); [Schultz et al., 1998](#_ENREF_28)). To identify putative modular proteins, we conducted a structural analysis of all proteins of *M. pneumoniae* for which a structure (or that of an ortholog protein) could be found in the PDB, as well as, a search for multi-domain proteins using Pfam (Table S2). Interestingly, 183 out of 689 proteins contain more than one structural/functional domain suggesting that they could be modular (Table S2). Long protein extensions could be misleading when assigning essentiality since this extension might be not essential, whilst the rest of the protein is or vice versa. To address this issue, we analyzed the 689 previously assigned ORFs ([Dandekar et al., 2000](#_ENREF_3)) using tBlastX in order to determine N- or C-termini extensions of at least 25 aa in length. Fourteen had putative extensions in their N- or C-termini, most of which are metabolic enzymes or ribosomal proteins.

**Essentiality of ncRNAs**

All ribosomal RNA and most tRNA genes are essential. Six tRNAs (MPNt4, -14,-15 -25, -26, -36) are non-essential most likely due to redundancy by other tRNAs, e.g. both MPNt25 and MPNt26 code for Ser. Interestingly, MPNt14 and MPNt15 coding for Arg tRNA and Gly tRNA are not essential whilst MPNt18 and MPNt17 also encoding for Arg tRNA and Gly tRNA are essential and fitness genes, respectively. Although there are three Leu tRNA genes (MPNt27, MPNt35 and MPNt36), only MPNt36 is non-essential; expression profiling indicates that MPNt36 is the most abundant (10 times more), suggesting that insertions in this tRNA can be tolerated during several rounds of cell division because high copy number per cell.

About 69% of the functionally non-assigned RNAs overlapped with other genes with 95% antisense to protein-coding genes (Table S2). The percentages of 79 essential antisense cis-RNAs inside essential, non-essential and fitness ORFs are 95%, 0%, and 5%, respectively. Only 11% of non-essential antisense cis- RNAs overlapped with essential ORFs. Excluding those parts that overlap with essential genes, we had a total numbers of 9 essential (5%), 18 fitness, and 162 non-essential ncRNAs. A BLAST alignment of intergenic ncMPNs (47 ncMPNs; IG in Table S2) with those from genomes of different species revealed that 47 of them are conserved across *M. pneumoniae* species and two of them are conserved in other bacteria (ncMPN007 and ncMPN322; seq-identity=63% and seq-identity=83%, respectively) (Figure S7). ncMPN322 is in antisense to *mpn348a* that possibly encodes for a conserved new protein of *M. pneumoniae* that could act as a toxin ([Liu et al., 2008](#_ENREF_17)) (Figure S5).

**Essentiality of 5´-UTRs and untranscribed regions**

Regarding 5´-UTR regions, the inserted minitransposons have an internal promoter that allows the transcription of downstream ORFs. This suggests that insertions in TSS upstream regions of essential ORFs can be tolerated as long as i) transcriptional regulation is not essential, ii) the 5´-UTR does not overlap, or iii) the 5´-UTR is proximal to an essential transcript in the same or opposite strand. We have studied the essentiality of 114 5´-UTRs longer than 70 bases, focusing on those that do not overlap with other genes (66 5´-UTRs; Table S2). We found three essential ORFs with an essential 5´-UTR (18% of the essential ORFs with a 5´-UTR longer than 70 bases). This suggests that some of the essential ORFs are strictly regulated. In contrast, any of the 36 5´-UTRs of non-essential genes is essential.

Finally, we looked into the chromosome regions that do not code for any RNA and that are longer than 100 bases (to avoid effects due to promoter regions considering an average approximation of 50 bases ([Shultzaberger et al., 2007](#_ENREF_29)), as well as the possibility of two opposite promoters side by side, and structural regions) (Table S3). After calculating expression values for intergenic regions by analysing RNAseq data we found 112 regions that are not transcriptionally active (log2CPKM<3, termed structural) (Table S3). Noteworthy, some of the structural regions are fitness at Thr7 suggesting that they can be essential under certain conditions. However by considering the most strict criteria (Thr41) 1 out 112 were essential (1%) and 7 fitness (6.2%). Thus 7.2% of structural regions could have a key role in chromosome structure, DNA duplication, or other cellular processes (Table S3). The two largest sequences without insertions comprise the bases 181 to 351 (171 bp in structural_2; Table S3) and 607941 to 608319 (379 bp in structural_209; Table S3). The search for DNA motifs in this region revealed the presence of eight equally distributed “TATTA” motifs that represent the DnaA boxes of *M. pneumoniae* (Yus & Serrano, manuscript in preparation). Moreover, a recent study of methylation in *M. pneumoniae* supports a role for this region in DNA replication ([Lluch-Senar et al., 2013](#_ENREF_18)). Interestingly, opposite to the site of the *oriC*, there is a structural region (structural_125; bases 410901 to 411097) with a common motif with the *oriC* region (5´-AT/AGCATTACC/AT/CATA-3´ P-value <2.2 x10^-16^) that could correspond to the replication termination site but it is non-essential for cell viability.

Taken together, this underscores that non-coding regions are important not only for a single category of elements, such as RNAs and that they need to be considered when deciphering a minimal bacterial genome.

# SUPPLEMENTARY FIGURES

**Figure S1. Study of essentiality by protein complexes and Pfam domains.** (A) Histogram of the percentage of essential and fitness ORFs (PFE) depending on the number of complexes in which the proteins are involved. (B) Percentage of essential and fitness ORFs (PFE) in protein complexes by COG categories. Functional categories are labeled: A, Post-translational modification, protein turnover, chaperones; B, Metabolism; C, DNA replication, recombination and repair D, Carbohydrate transport and metabolism; E, Transcription; F, Translation, ribosomal structure and biogenesis; G, Energy production and conversion; H, Amino acid transport and metabolism; I, Function unknown; J, Defense mechanisms; K, Cell division and chromosome partitioning; L, Cell envelope biogenesis, outer membrane. C) The histogram represents the percentage of essential and fitness ORFs (PFE) depending on the number of domains for the proteins involved in complexes (green) or not (grey). D) Essentiality in the DNA replication and repair processes. The essential ORFs involved in the different steps of these processes are indicated with an asterisk (*) and, fitness ORFs with a delta (#) (Table S2).

**Figure S2. Overexpression of independent domains of putative modular protein.** Domains of putative modular proteins were expressed in *E. coli* BL21(DE3) pLysS and *E. coli* BL21 pRARE strains. A) SDS-PAGE gel of the soluble fraction of the cell lysate overexpression of: MPN241a in *E. coli* BL21(DE) pLys (lane1) and *E. coli* BL21(DE) pRARE respectively ( lane 2); lanes 3 and 4 correspond to the soluble fractions of the strains overexpressing MPN623a (no protein detected; neither domain b; data not shown) and lanes 5 and 6 show protein MPN683a overexpressed in both *E. coli* strains. B) Schematic representation of the genomic regions that comprises the domains of the proteins that have been expressed. The color of the boxes in panel B corresponds with the colors of the boxes in panel A. Transposon insertions are shown as black vertical bars.

**Figure S3. Conservation and Essentiality of putative smORFs**. A) The circular diagram represents the percentage of the putative smORFs identified in the *in silico* translation of the genome, that are conserved in different categories shown in the legend. The histogram represents the percentage of essential (E), non-essential (NE), fitness (F) and non-determined (ND) categories for the smORFs in function of the conservation (in purple, smORFs conserved in bacteria; and in light blue, smORFs conserved in *M. pneumoniae* strains). B) Western Blots of the elution fractions of molecular weight exclusion chromatographies of different *M. pneumoniae* strains expressing the proteins MPN060a, MPN155a and MPN474a fused to a TAP-tag. Red lines indicate the experimental molecular weight assigned to the elution volume of the correspondent fraction.

**Figure S4. Functionality of smORFs.** A) Putative toxin-antitoxin operon in *M. pneumoniae*. The upper panel represents information obtained by RNAseq for the plus (grey and black dots) and the minus (purple dots). Coding regions of the three ORFs are indicated by arrows: MPN347a new identified protein by MS (first dark grey arrow); HP? a putative ORF, whose protein has not been identified by MS (clear grey arrow) and MPN348a coding for the putative doc toxin (black arrow). The purple arrow indicates an antisense RNA that is coded by the minus strand. B) The protein alignment that allowed the annotation of MPN348a. This protein belongs to the toxin-antitoxin system “Death on cure” family ([Garcia-Pino et al., 2008](#_ENREF_6); [Lehnherr et al., 1993](#_ENREF_15)). It shows a high sequence homology with the DOC protein of *M. pneumoniae* FH strain. Blue residues indicate the conserved residues of the active center of the toxin (predicted to have the catalytic activity). In yellow is shown a stop codon (TAA) that is present in *M.pneumoniae* M129 but in *M. pneumoniae* strain FH is a GAA codon coding for glutamic acid. In red, is shown the L encoded by TGG that could act as alternative start codon in *M. pneumonie*. Second alignment shows the conservation across different bacteria of the dic motif coding for the protein active center C) MPN155a. Location of *mpn155a* in the genome of *M. pneumoniae* and sequence alignment using ClustalW2 ([Larkin et al., 2007](#_ENREF_14)) with the YlxR protein from *S. pneumoniae* ([Osipiuk et al., 2001](#_ENREF_22)).

**Figure S5. Transposon insertion bias by GC content.** A) The graph indicates the density of insertions in a 200 size window versus the GC content considering the non-essential and non-repetitive regions of *M. pneumoniae* genome. Red dots are the values for insertions in pMTnTetM438 sample and black dots for pMT85 sample. Black line shows the relative abundance of windows of the same length with a particular GC content in the genome. B) Transposon insertion bias by sequence. The densities of insertions in the 256 putative quadruplet sequences are represented in function of GCAT score (A,T=1, GC=2). Interestingly, despite quadruplets with high AT content show higher frequencies of insertions there are some quadruplets having GC at the two fist bases that show also a bias (red sequences). C) Correlation of frequencies of quadruplets in smORFs versus conventional ORFs. The graph indicates that the frequencies are similar in both genome categories confirming that despite there is a bias specific of quadruplet sequence it is affecting equally to smORFs and ORFs.

**Figure S6. Essential metabolome of *M. pneumoniae***. The essential genes involved in the different reactions of the experimentally defined metabolome of *M. pneumoniae* are shadowed in yellow and, fitness in pink; non-essential genes are not marked.

**Figure S7. Growth curve of *M. pneumoniae* M129 strain.** Black dots show the rations of absorbance measured at 425nm and 560 nm wave lengths. The ratio gives an indication of the medium pH that decreases in *M. pneumoniae* growth. It is one of the methods currently used to estimate *M. pneumoniae* growth curve. 10 time-points corresponding to the lag and log phases of growth curve were studied by RNAseq: 1 hour (h), 2h, 6h, 8h, 10h, 12h, 24h, 36h, 48h, and 72h..

# **Figure S8. Comparative study of Essentiality.** A) In total 122 genes in *M. pneumonia (mpn)* and 120 in *M. genitalium* (mg) were found to be orthologs to the 129 genes suggested to be the minimal core for Mollicutes by Grosjean et al ([Grosjean et al., 2014](#_ENREF_9)). We show the percentage of genes that are E, NE and F for the ortholog genes. B) Comparative essentiality analysis between *M. pneumoniae* and *M. genitalium*. 444 pairs of one-to-one ortholog genes between *M. pneumoniae* and *M. genitalium* were extracted from firmNOG of eggNOG ver 4.0 ([Powell et al., 2014](#_ENREF_25)). Since there is no F category for *M. genitalium*, we assigned the *M. pneumoniae* F genes to either E (F==E) or NE (F==NE). The results do not change regardless to which group (essential (F==E; left stacked bar) or non-essential (F==NE; right stacked bar) the fitness genes were assigned (Table S11).

# LEGEND OF SUPPLEMENTARY TABLES

**Table S1A: The** **essential gold set** is composed of the RNA polymerase subunits, the unique sigma 70 factor, the tRNA synthases, the DNA polymerase complex, the ribosomal RNA ([Yus et al., 2009](#_ENREF_33)). **Table S1B: The Non-essential gold set** is composed of a group of *M. pneumoniae* genes that are not found in the closely related species *M. genitalium*. Columns: 1) MPN number , 2) function of the ORFs, 3) ORF size after removing 10% of the gene length from the Nter and Cter in bp, 4) number of insertions found in the region with Thr7, 5) and 6) the E and NE probabilities for Thr7, 7) the number of insertions found in the region with Thr41, 8) and 9) the E and NE probabilities for Thr41 and 10) and 11) the essentiality assigned for Thr7 and Thr41, Essential genes (P_E_ >0 and P_NE_ =0; E), Non-essential (P_E_ =0 and P_NE_ >0) and fitness genes (P_E_ >=0 and P_NE_ =>0). Numbers were rounded to two decimals.

Columns 12 to 22 are the same than columns 1 to 11 but for the non-essential gold set.

**Table S2A.** **Essentiality ORFs.** **i)** **A)** **Genomic Information** (Colums 1 to 10). Column 1 (ORF), Protein coding genes are termed MPN, genes corresponding to ribosomal RNAs are termed MPNr, genes corresponding to tRNAs are indicated as MPNt. The ORFs of newly identified proteins are assigned with the name of the previous gene followed by an “a”. Columns 2-5 indicate respectively: the identifier of the protein in the new protein database; the strandwhere "+" is forward and "-" is reverse; the protein name and the function of the ORF, respectively. Column 6 indicates the COG category of the ORF (A, Membrane Proteins of unknown function; C, Energy production and conversion, Coenzyme metabolism; D, Cell division and chromosome partitioning; E, Amino acid transport and metabolism; F, Nucleotide transport and metabolism; coenzyme; G, Carbohydrate transport and metabolism, H, Coenzyme metabolism; I, Lipid metabolism; J Translation, ribosomal structure and biogenesis; K, Transcription; L, DNA replication, recombination and repair; M, Cell envelope biogenesis, outer membrane; N, Cell motility and secretion; O, Post-translational modification, protein turnover, chaperones; P, Inorganic ion transport and metabolism; R, General function prediction only; S, Function unknown; T, Signal transduction mechanisms; U, Intracellular trafficking, secretion and vesicular transport; and V, Defense mechanisms). Column 7 (TSS), corresponds to the genome position of the transcriptional start site. Dotted lines indicate that the TSS could not be experimentally assigned. Column 8 (TTS), indicates the genome position for the transcription termination site. Column 9 (TSC), indicates the genome position of the translational start codon after re-annotation and column 10 (Stop), shows the genome position corresponding to the first base of the stop codon.

**B) Essentiality study of ORFs** and ncRNAs (columns 10 to 25). Column 11 shows the length of the regions considered for essentiality study (ORF length less 20% of its size taken from the N and C-termini) for protein coding genes, the length of the functional part of rRNA and tRNA. Column 12 indicates the number of insertions found by HITS for Thr7. Columns 13 and 14 indicate, for every ORF, the probability of being E (PE) and essential (PNE) for Thr7, respectively. Column 15, the number of insertions found by HITS for Thr41. Columns 16 and 17 indicate, for every ORF, the probability of being E (PE) and essential (PNE) for Thr41, respectively. Column 18 and 19 show the essentiality assigned using the criteria of probabilities as described in Experimental procedure section (E, essential; NE, non-essential; NE*, non-essential containing repetitive regions; and F, fitness) for Thr 6 and Thr41, respectively. Column 20 indicates the essentiality assigned after confirming the insertions by PCR and sequencing of the amplified fragment. The clone was then isolated from the pool and grown (E, essential; NE, non-essential; F, fitness; Experimental Procedures). Column 21 indicates the name of the gene that is overlapping with a non-coding RNA (ncMPNs). Column 22, A overlap in antisense; S overlap in sense, IG intergenic region. When a ncRNA overlaps with two ORFs, the names of the two genes are indicated. Columns 22 to 24 indicate the overlapping region of the ncMPN with the respective ORF: 5’ region (column 22 shows the region of the ORF comprising one third of the gene length corresponding to N-terminus); Middle region (column 23) and the 3’ region (column 24; Cterminus of the ORF). Part=partial overlap; All=complete overlap. Column 25, indicates the essentiality of overlapping ORF.

**C)** **Summary of Essentiality assigned to Pfam domains.**Comparison of the essentiality assigned to Pfam domains against essentiality of the corresponding ORF. Column 26, Interpro identities; Column 27, accession number of Pfam domain; Columns 28 and 29, essentiality based on P_E_ and P_NE_ for Thr7 and Thr41, respectively; Column 30, number of Pfam domains identified in the protein; Column 31 the putative modular proteins (indicated with a “x”).

**D) Essentiality of 5´-UTRs**. In column 32, the size of the 5´-UTR region associated to the ORF. Column 33 and 34 show the number of insertions in 5´-UTR; 35 and 36: essentiality for Thr7 and Thr41, respectively. 37 indicates the essentiality of the corresponding ORF. Column 38 shows the names of the ORFs that are overlapping with a 5´-UTR.

**Table S3. Structural Regions.** This table contains the regions of the genome longer than 100 bp that are not transcriptionally active. Column 1, the name of the region; column 2, start of the genomic region; column 3, end of the genomic region; column 4, the length of the region in bp; column 5, the number of insertions identified in the region by HITS with Thr7; columns 6 and 7, the probability of the region to be essential (P_E_) or non-essential (P_NE_) for Thr7, respectively; and columns 8 to 9 the same than 5 to 7 columns but for Thr41. 11 and 12 the essentiality assigned in Thr7 and Thr41, respectively. The essentiality groups are defined as for Table S1.

**Table S4. Study of effect of ncRNAs in the expression of complementary ORF.** Columns 1 to 4 show Pearson correlations and CLR_scores of ncRNA and complementary ORF along the growth curve. Columns 5 to 8 show Thr41 essentiality of ncRNAs (column 6) versus the percentage of essential ORFs that correlate (column 7) and anticorrelate (column 8) along growth curve.

**Table S5A. smORFs in *M. pneumoniae* proteome determined experimentally**. **A) Genomic context.** Column 1 indicates the name of the smORF (<100aa). New proteins that are more conserved at protein level than aa level are marked with an “α” symbol. SmORFs studied by TAP-tag marked by “&” symbol and the expressed ones with an asterisk “*”. Column 2 describes the function of the encoded protein when it is known. Column 3 shows the results of Blast alignment using protein data from NCBI. The cellular organelle represents the higher rank of conservation. Bacteria it indicates that the protein is conserved at superkingdom level. Mycoplasma, when it is conserved across different species. *M. pneumoniae*, when the protein is conserved across different Mycoplasma strains. Column 4 shows assigned essentiality.

**B) Gene Expression.** Columns 5 and 6: Transcription levels of different genes determined by RNA-seq at exponential (6h) and stationary (96) phases of growth by using data of non-fractionated RNA previously described by Yus E., et al. ([Yus et al., 2012](#_ENREF_32)). Columns 7 and 8, new samples obtained to study the levels of expression along growth curve (See sections of RNAseq described above). Represented values correspond to the log2 of CPKM.

**C) Complex formation**. Column 9 and 10 indicate the number of unique peptides (Nr UP) identified for each protein in two independent size exclusion chromatography experiments (Column 9: cell extract treated with DNaseI and column 10: cell extract treated with DNAseI and RNaseIII). In columns 11 and 12 are indicated the expected and experimental molecular weights (Mw) of the proteins, respectively.

**D) DNA binding experiments.** The numbers in the different columns indicate the unique peptides detected for each protein in each experiment: Column 13: DNA cellulose columns; Column 14: elution with 1M NaCl Column 15: elution with RNA.

**E) Sucrose cushion** to isolate chromatin.

**F)** **SDS Gels.** In column 17, "x" indicates that the protein has been detected by MS from bands in a Bis-Tris SDS gel loaded with total protein extract of *M. pneumoniae*. In column 18, "x" indicates that the protein has been detected by MS from bands in a glycine SDS gel loaded with total protein extract of *M. pneumoniae*. In column 19, "x" indicates that the protein has been detected by MS from cell lysates at two time points: 6 hr and 96 hr. Column 20 indicates the number of unique peptides identified by MS. Unique peptides are those that were identified by MS and are exclusive for that specific protein.

**Table S6. A) SEC_MS Cell extract treated with DNAse I.** Elution profiles of proteins from *M. pneumoniae* total protein extract, purified by molecular weight exclusion chromatography. C to AA columns indicate the molecular weight of the different elution fractions analyzed by MS. For each ORF and each fraction (molecular weight) of the purification we have calculated the amount of protein by MS (the area of the three best identified peptides). **B) SEC_MS Cell extract treated with DNAse I and RNAse.** Elution profiles of proteins from *M. pneumoniae* total protein extract, purified by molecular weight exclusion chromatography. C to AA columns indicate the molecular weight of the different elution fractions analyzed by MS. For each ORF and each fraction (molecular weight) of the purification we have calculated the amount of protein by MS (the area of the three best identified peptides).

**Table S7.** Results for sequencing and genome mapping in the different samples derived from transformation with both vectors pMT85 (TnGm) and pMTnTetM438 (TnTc) . Cells were passaged twice and incubated for a total of 12 days.

**Table S8. Minitransposon insertion sites.** We show the genome position and the number of reads for each minitransposon (pMT85 (TnGm) and pMTnTetM438 (TnTc)) insertion after growing cells for 12 days. We applied the Thr7 filterThe last two columns show the the genome position of the insertion and the results of merging the datasets for both transposons after applying the Thr7 or Thr41 filters.

**Table S9.** Primer sequences used for cloning of smORFs in TAP-tap experiments. Also, the sequences of the primers for the expression of modular proteins are shown in the table.

**Table S10. Thresholds to identify DNA/RNA binding proteins.** The table indicates the area under the ROC curve (AUC) for each DNA/RNA binding experiment: NaCl elution and RNA elution. Also, we show the values of the thresholds used for each experiment as well as the true positives (TP) versus false positive (FP) ratios (Supplementary Materials)

# **Table S11. A) Comparative with the translational minimal apparatus for Mollicutes predicted by Grosjeans et al (**[**Grosjean et al. 2014**](#_ENREF_9)**). B) Comparative between the one to one ortolog genes from *M. genitalium* (MG) and *M. pneumoniae* (MPN*).***

# **REFERENCES**

Bentley, D.R., Balasubramanian, S., Swerdlow, H.P., Smith, G.P., Milton, J., Brown, C.G., Hall, K.P., Evers, D.J., Barnes, C.L., Bignell, H.R.*, et al.* (2008). Accurate whole human genome sequencing using reversible terminator chemistry. Nature *456*, 53-59.

Christen, B., Abeliuk, E., Collier, J.M., Kalogeraki, V.S., Passarelli, B., Coller, J.A., Fero, M.J., McAdams, H.H., and Shapiro, L. (2011). The essential genome of a bacterium. Molecular systems biology *7*, 528.

Dandekar, T., Huynen, M., Regula, J.T., Ueberle, B., Zimmermann, C.U., Andrade, M.A., Doerks, T., Sanchez-Pulido, L., Snel, B., Suyama, M.*, et al.* (2000). Re-annotating the Mycoplasma pneumoniae genome sequence: adding value, function and reading frames. Nucleic acids research *28*, 3278-3288.

Eraso, J.M., Markillie, L.M., Mitchell, H.D., Taylor, R.C., Orr, G., and Margolin, W. (2014). The highly conserved MraZ protein is a transcriptional regulator in Escherichia coli. J Bacteriol *196*, 2053-2066.

Faith, J.J., Hayete, B., Thaden, J.T., Mogno, I., Wierzbowski, J., Cottarel, G., Kasif, S., Collins, J.J., and Gardner, T.S. (2007). Large-scale mapping and validation of Escherichia coli transcriptional regulation from a compendium of expression profiles. PLoS biology *5*, e8.

Garcia-Pino, A., Christensen-Dalsgaard, M., Wyns, L., Yarmolinsky, M., Magnuson, R.D., Gerdes, K., and Loris, R. (2008). Doc of prophage P1 is inhibited by its antitoxin partner Phd through fold complementation. The Journal of biological chemistry *283*, 30821-30827.

Ghaemmaghami, S., Huh, W.K., Bower, K., Howson, R.W., Belle, A., Dephoure, N., O'Shea, E.K., and Weissman, J.S. (2003). Global analysis of protein expression in yeast. Nature *425*, 737-741.

Glass, J.I., Assad-Garcia, N., Alperovich, N., Yooseph, S., Lewis, M.R., Maruf, M., Hutchison, C.A., 3rd, Smith, H.O., and Venter, J.C. (2006). Essential genes of a minimal bacterium. Proceedings of the National Academy of Sciences of the United States of America *103*, 425-430.

Grosjean, H., Breton, M., Sirand-Pugnet, P., Tardy, F., Thiaucourt, F., Citti, C., Barre, A., Yoshizawa, S., Fourmy, D., de Crecy-Lagard, V.*, et al.* (2014). Predicting the minimal translation apparatus: lessons from the reductive evolution of mollicutes. PLoS genetics *10*, e1004363.

Hutchison, C.A., Peterson, S.N., Gill, S.R., Cline, R.T., White, O., Fraser, C.M., Smith, H.O., and Venter, J.C. (1999). Global transposon mutagenesis and a minimal Mycoplasma genome. Science *286*, 2165-2169.

Kimura, S., and Suzuki, T. (2010). Fine-tuning of the ribosomal decoding center by conserved methyl-modifications in the Escherichia coli 16S rRNA. Nucleic acids research *38*, 1341-1352.

Kuhner, S., van Noort, V., Betts, M.J., Leo-Macias, A., Batisse, C., Rode, M., Yamada, T., Maier, T., Bader, S., Beltran-Alvarez, P.*, et al.* (2009). Proteome organization in a genome-reduced bacterium. Science *326*, 1235-1240.

Kumar, A., Seringhaus, M., Biery, M.C., Sarnovsky, R.J., Umansky, L., Piccirillo, S., Heidtman, M., Cheung, K.H., Dobry, C.J., Gerstein, M.B.*, et al.* (2004). Large-scale mutagenesis of the yeast genome using a Tn7-derived multipurpose transposon. Genome research *14*, 1975-1986.

Larkin, M.A., Blackshields, G., Brown, N.P., Chenna, R., McGettigan, P.A., McWilliam, H., Valentin, F., Wallace, I.M., Wilm, A., Lopez, R.*, et al.* (2007). Clustal W and Clustal X version 2.0. Bioinformatics *23*, 2947-2948.

Lehnherr, H., Maguin, E., Jafri, S., and Yarmolinsky, M.B. (1993). Plasmid addiction genes of bacteriophage P1: doc, which causes cell death on curing of prophage, and phd, which prevents host death when prophage is retained. Journal of molecular biology *233*, 414-428.

Letunic, I., Doerks, T., and Bork, P. (2012). SMART 7: recent updates to the protein domain annotation resource. Nucleic acids research *40*, D302-305.

Liu, M., Zhang, Y., Inouye, M., and Woychik, N.A. (2008). Bacterial addiction module toxin Doc inhibits translation elongation through its association with the 30S ribosomal subunit. Proceedings of the National Academy of Sciences of the United States of America *105*, 5885-5890.

Lluch-Senar, M., Luong, K., Llorens-Rico, V., Delgado, J., Fang, G., Spittle, K., Clark, T.A., Schadt, E., Turner, S.W., Korlach, J.*, et al.* (2013). Comprehensive methylome characterization of Mycoplasma genitalium and Mycoplasma pneumoniae at single-base resolution. PLoS genetics *9*, e1003191.

Lluch-Senar, M., Querol, E., and Pinol, J. (2010). Cell division in a minimal bacterium in the absence of ftsZ. Mol Microbiol *78*, 278-289.

Meyer, P.E., Lafitte, F., and Bontempi, G. (2008). minet: A R/Bioconductor package for inferring large transcriptional networks using mutual information. BMC bioinformatics *9*, 461.

Mulder, N.J., Apweiler, R., Attwood, T.K., Bairoch, A., Bateman, A., Binns, D., Biswas, M., Bradley, P., Bork, P., Bucher, P.*, et al.* (2002). InterPro: an integrated documentation resource for protein families, domains and functional sites. Briefings in bioinformatics *3*, 225-235.

Osipiuk, J., Gornicki, P., Maj, L., Dementieva, I., Laskowski, R., and Joachimiak, A. (2001). Streptococcus pneumonia YlxR at 1.35 A shows a putative new fold. Acta crystallographica Section D, Biological crystallography *57*, 1747-1751.

Osterman, A.L., and Gerdes, S.Y. (2008). Microbial Gene Essentiality, Vol 416 (Totowa, NJ: Humana Press Inc).

Pich, O.Q., Burgos, R., Planell, R., Querol, E., and Pinol, J. (2006). Comparative analysis of antibiotic resistance gene markers in Mycoplasma genitalium: application to studies of the minimal gene complement. Microbiology *152*, 519-527.

Powell, S., Forslund, K., Szklarczyk, D., Trachana, K., Roth, A., Huerta-Cepas, J., Gabaldon, T., Rattei, T., Creevey, C., Kuhn, M.*, et al.* (2014). eggNOG v4.0: nested orthology inference across 3686 organisms. Nucleic acids research *42*, D231-239.

Prasad, S.C., and Dritschilo, A. (1992). High-resolution two-dimensional electrophoresis of nuclear proteins: a comparison of HeLa nuclei prepared by three different methods. Analytical biochemistry *207*, 121-128.

Punta, M., Coggill, P.C., Eberhardt, R.Y., Mistry, J., Tate, J., Boursnell, C., Pang, N., Forslund, K., Ceric, G., Clements, J.*, et al.* (2012). The Pfam protein families database. Nucleic acids research *40*, D290-301.

Schultz, J., Milpetz, F., Bork, P., and Ponting, C.P. (1998). SMART, a simple modular architecture research tool: identification of signaling domains. Proceedings of the National Academy of Sciences of the United States of America *95*, 5857-5864.

Shultzaberger, R.K., Chen, Z., Lewis, K.A., and Schneider, T.D. (2007). Anatomy of Escherichia coli sigma70 promoters. Nucleic acids research *35*, 771-788.

Taniguchi, Y., Choi, P.J., Li, G.W., Chen, H., Babu, M., Hearn, J., Emili, A., and Xie, X.S. (2010). Quantifying E. coli proteome and transcriptome with single-molecule sensitivity in single cells. Science *329*, 533-538.

Wodke, J.A., Puchalka, J., Lluch-Senar, M., Marcos, J., Yus, E., Godinho, M., Gutierrez-Gallego, R., dos Santos, V.A., Serrano, L., Klipp, E.*, et al.* (2013). Dissecting the energy metabolism in Mycoplasma pneumoniae through genome-scale metabolic modeling. Molecular systems biology *9*, 653.

Yus, E., Guell, M., Vivancos, A.P., Chen, W.H., Lluch-Senar, M., Delgado, J., Claude Gavin, A., Bork, P., and Serrano, L. (2012). Transcription start site associated RNAs in bacteria. Molecular systems biology *8*, 585.

Yus, E., Maier, T., Michalodimitrakis, K., van Noort, V., Yamada, T., Chen, W.H., Wodke, J.A., Guell, M., Martinez, S., Bourgeois, R.*, et al.* (2009). Impact of genome reduction on bacterial metabolism and its regulation. Science *326*, 1263-1268.
