# Supplementary material for: Defining a minimal cell: essentiality of small ORFs and ncRNAs in a genome-reduced bacterium
Source: Mol Syst Biol. 2015 Jan 21;11(1):780. doi: 10.15252/msb.20145558 (PMC4332154; doi:10.15252/msb.20145558)
Supplement: Supplementary file 22 [file msb0011-0780-sd22.pdf]

Defining a minimal cell: essentiality of small ORFs and ncRNAs in a genome-reduced bacterium

Maria Lluch-Senar, Javier Delgado, Wei-Hua Chen, Verónica Lloréns-Rico, Francis J. O'Reilly, Judith A.H. Wodke, E. Besray Unal, Eva Yus, Sira Martínez, Tony Ferrar, Ana Vivancos, Arne G. Schmeisky, Jörg Stülke, Vera Van Noort, Anne-Claude Gavin, Peer Bork, and Luis Serrano

Corresponding author: Luis Serrano, Centre for Genomic Regulation CRG

Review timeline:

Submission date:	07 July 2014
Editorial Decision:	26 August 2014
Revision received:	06 November 2014
Editorial Decision:	04 December 2014
Revision received:	15 December 2014
Accepted:	18 December 2014

Editor: Maria Polychronidou

Transaction Report:

(Note: With the exception of the correction of typographical or spelling errors that could be a source of ambiguity, letters and reports are not edited. The original formatting of letters and referee reports may not be reflected in this compilation.)

1st Editorial Decision 26 August 2014

Thank you again for submitting your work to Molecular Systems Biology. We have now heard back from the three referees who agreed to evaluate your manuscript. As you will see from the reports below, the referees acknowledge that the presented findings are potentially interesting. However, they raise a series of concerns, which should be carefully addressed in a revision of the manuscript.

In particular, reviewer #2, points out that in order to convincingly support the findings on essential smORFs and ncRNAs, it is essential to repeat the analysis with a significantly larger number of mutants. Please note that we have also circulated the reports to all reviewers as part of our 'pre-decision cross-commenting' policy. During this process, reviewer #3, who raised similar concerns regarding the size of the mutant library, mentioned that s/he agrees with reviewer #2 that the analysis needs to be repeated on a larger sample. As you will see below ("NOTE from the editor"), we have further consulted with reviewer #2, to confirm that these additional analyses can be performed within the scope of a major revision.

Reviewer #1:

The paper submitted for publication in Mol Syst Biology by Lluch-Senar et al is entitled "Defining a minimal cell: essentiality of small ORFs and ncRNAs in a genome-reduced bacterium". It is submitted as a short publication (report) with 4 composite figures and a substantial supplementary material.
This study represents a significant step in the efforts for building a minimal cell. It reveals an unexpected degree of complexity indicating that there are a number of non-coding RNAs that are

© European Molecular Biology Organization

essential. In addition, it points to the important roles played in a minimal cell by small proteins.
Note: I numbered the page of the manuscript taking the cover page as page #1.

General points :
ï One of the questions around genome minimization is to determine the size of a minimal genome. The authors should have the means based on the present study to give at least a range of gene number/size of genome for a minimal genome starting with M. pneumoniae chassis.
ï For the translational machinery, a recent study published in PLoS Genetics by Grosjean et al suggested that the minimal protein machinery in Mollicutes would include 129 proteins/genes, a set slightly smaller than the translational gene set reported for M. pneumoniae in the same study. Are these 129 proteins/genes found as essential in the present study?
ï M. genitalium and M. pneumoniae, although colonizing different mucosal surfaces or producing distinct human diseases, are very closely related bacteria. In a previous study by Glass et al (2006), it was indicated that "... some M. genitalium orthologs of nonessential M. pneumoniae genes are in fact essential". Does the present study confirm this suggestion? In fact, the results obtained in this 2006 paper could be compared with the results obtained here.

Minor comments on the main text and figures:
p 2 : I would remove ´ an accurate view of a minimal genome ª from the abstract as it is not clear what it really means ;

p 3:
- "...and NE (20 genes) strains": I believe that the authors mean "clones" rather than "strains"
- "C. crescentus", the name of the genus needs to be provided as it was not cited before in the manuscript;

p 4:
- "showed autonomous folding"; this claim does not seem to be supported by the data shown Fig. S2. Please clarify.
- the origin of replication of bacterial chromosomes is usually abbreviated as oriC, and not as "ORI";

p11: "Thermotoga" instead of "Termotoga"

Figure 1: the essentiality genome map is too small, it is not possible to view the location of the insertions nor the gene names. It should be provided supplementary material. In Fig 1A, affinity is misspelled.

Minor comments on Supplementary data :

ï It would be useful for future studies to include a high resolution map of the transposon insertions and/or a table with all insertion positions

ï Most of the figures in the supplementary materials should be enlarged for improving the manuscript readability;

ï p 7 : concerning the expression of domains of putative modular proteins, was it necessary to avoid the UGA codons (coding for Trp in mycoplasmas)? This needs to be specified

ï p 9: "Glycine" instead of "Glicine"

ï p 10 and elsewhere : misspelling, check: "M. pneumoniae" instead of "M. pneumoniae"

ï p 12 : a problem in the pdf production after ´with 0.1% FA in 60 min at a flow of 0.3 ª

ï p 20 : ´ In contrast, only 9 % of non-essential antisense cis- RNAs overlapped with essential ORFs ª. How is it possible to conclude that a ncRNA is not essential when it is antisense from an essential CDS?

ï p 20 and 33: ´ two of them [ncRNA] are conserved in other bacteria (ncMPN007 and ncMPN322;

e-value < 0.005) [a]. It is not clear if these regions have been documented as being transcribed in other bacteria such as H. pylori. In addition, instead of provided an e-value from a Blast analysis, it would be more meaningful to provide a percentage of identity indicating the length of the aligned sequences.

Reviewer #2:

Note that I have provided a Word file that includes some figures I have generated to better show the problem with this manuscript.

The manuscript by Maria Lluch-Senar and colleagues is a bold effort to determine what are the essential genetic elements in a near minimal bacterium. Over the last 6 years or so, this team has published a dazzling series of papers characterizing the atypical bacterium, Mycoplasma pneumoniae, from a systems biology perspective. Because this organism, at only 860 kb and 800 genes, is already close to being a minimal bacterial cell, determination of what genes and regions of genes are essential and what is non-essential is of great interest. Since the days of the Max Delbruck's phage school in the 1930's it has been the dream of biologists to use a reductionist approach to understanding how life works by identifying and determining how each essential element in a living cell functions. The aim of this project is to identify, and to some extent characterize all the essential genetic material in a cell by marrying proteomics, and transcriptomics data with the knowledge of what genetic material can be disrupted using transposon bombardment. The strategy employed by Lluch-Senar and colleagues is much like the approach taken by the team of Stanford microbiologist Lucy Shapiro to identify the essential genes in Caulobacter crescentus in 2011 that was published in Molecular Systems Biology. The Stanford team combined hyper-saturated transposon mutagenesis coupled with high-throughput sequencing. The protein coding genes, non-coding RNAs and other un-translated genetic elements such as origins of replication and transcriptional control regions that were not hit by transposons were defined as essential.

In an absolute sense, transposon bombardment only defines the non-essential regions of the genome. Even then, it is possible if the experiment is not set up properly to incorrectly label an essential gene as non-essential. For instance, a gene encoding a protein for which there are many copies in a cell, but for which only one copy is essential could be disrupted by a transposon and still result in cells that might survive 10 or more cell divisions. Thus, after the transposon reaction it is necessary to culture cells long enough that the entire pool of proteins initially present in transposon disrupted cell is exhausted. Similarly, as reported in both the Stanford Caulobacter paper and the Barcelona Mycoplasma manuscript being reviewed, disruption of some genes results in cells that have impaired growth phenotypes, called Fitness genes. To determine the essential regions of a genome one must make several assumptions about the transposon bombardment process:
ï Transposon insertions are completely random
ï The number of independent transposon mutants being analyzed is great enough that there is a high probability that all non-essential targets are hit.
The Stanford Caulobacter project used a pool of ~800 thousand viable Tn5 transposon insertion mutants. Tn5 transposition is almost random as was demonstrated by Fred Blattner and colleagues (Systematic Mutagenesis of the Escherichia col Genome. 2004. Journal of Bacteriology 186(15):4921.

In the EMBL/CRG Mycoplasma pneumoniae paper under review the story is quite different. First, Tn4001 insertions are not random, as was shown in other studies using Tn4001 in Mycoplasma genitalium and Mycoplasma pneumoniae (Glass et al. 2006, and Hutchison et al 1999). Some genes were hit many more times than others. In Glass et al. and also in French et al.'s study of essentiality of Mycoplasma pulmonis, several thousand individual mutants were isolated and the locations of each transposon insertion were determined. In many cases, for example with M. genitalium gene MG_414, several hundred of the ~4000 Tn4001 mutants analyzed had the transposon insertion at the same base of that gene. Second, and more important, not enough mutants were analyzed to make accurate predictions about what genes, especially small genes, are essential. The EMBL/CRG Mycoplasma pneumoniae paper stated that they obtained a pre-existing pool of 2976 Tn4001 mutants first reported on in 2006 and later in 2007 by Halbedel and Stulke. That said, consider the likelihood a non-essential genetic element will be disrupted.

A 2007 paper by Halbedel and Stulke, which was offered by the EMBL/CRG Mycoplasma pneumoniae paper authors cited to explain the probability of identifying specific fractions of the total set of non-essential genes and other genetic elements offers this formula: The probability that a mutation in a gene of interest comprised of g base pairs will be found in a given collection of transposon mutants. Here n is the number of mutants, l is the non-essential genome size, and P is the probability.

$$n = \log (1-P) / \log(1-(g/l))$$

It is assumed that given that all genes in M. genitalium have orthologous genes in M. pneumoniae, and that the genes present in M. pneumoniae but absent in M. genitalium are non-essential. Thus the 236 kb of DNA in M. pneumoniae (816 kb) not in M. genitalium (580 kb) are non-essential. Additionally, Glass et al. disrupted another 100 M. genitalium genes (comprising 112 kb) and others have found another 8 kb of non-essential M. genitalium genes. So the non-essential M. pneumoniae genome is at least 356 kb. Assuming the minimum small RNA coding gene one is searching for is 100 bp, then one has only a 57% chance of disrupting that gene. If the minimum target is a 100 amino acid protein, then there is a 92% chance it will be disrupted. If the minimum target were a 100 base pair gene or noncoding RNA then the probability is on a bit over 50% it would be disrupted and categorized as not essential.

About that same point, the authors state: "With this number of mutants, the probability of finding a desired mutant in the library is 99.999%." This is almost the same sentence in the 2006 paper by Halbedel et al. where they calculated the probability of finding a mutant among their pool of 2976 mutants that had a disrupted hprK gene. The Halbedel sentence was: "With this number of mutants, a hprK mutant is included in the library with a probability of 99.999%." The hprK ORF is 936 nucleotides in length. By my calculation, using current reports of the non-essential genome in M. genitalium which indicates the non-essential genome is 356 Kb, the probability today would be 99.996% that that mutant would be found; however, for small transcripts the probability is much lower. For instance, the likelihood a 75 bp gene would be hit is only 47%. As shown in this chart, to have 99% confidence that a gene was hit among the 2976 M. pneumoniae mutants analyzed, the minimum target size would have to be about 550 base pairs.

The authors of this paper did state that they mapped of 34,825 unique mini-transposon insertions at a low resolution (-9 bp), but this number is the result of the 2976 mutants in the pool of mutants provided by Halbedel and Stulke being cultured. DNA was extracted from cultures comprised of those 2976 mutants. Those mutants, because different transposons affect mutant growth rates differently, will not be equimolar. Additionally, depending on how long those mutants were cultured, there could be genomes remaining of cells that had non-viable transposon insertions that were swept up by Haldebel and Stulke when they made their mutant library. This could explain the surprising incidence of gene disruptions in ORFs that based on our knowledge of bacterial physiology would have to be essential. Hutchison et al., in their 1999 paper found evidence of transposon hits in a number of genes such as DNA polymerases and tRNA synthetases that are known to be essential. Note that they did not isolate any transposon mutants, rather they isolated DNA from a pool of Tn4001 mutants in M. genitalium and M. pneumoniae, fragmented the genomic DNA, treated it with DNA ligase to circularize the fragments, and then did inverse PCR using outward facing Tn4001 primers to amplify transposon-genome junctions.

Quite simply, the conclusions reached by the EMBL/CRG group about essential small ORFs and non-coding RNAs are not supported given they only analyzed a few less than 3000 mutants. They probably should have analyzed at least 100 thousand clones, and probably several times that amount. It is likely that they have greatly overestimated the number of small ORFs and non-coding RNAs that are essential. There is no problem with the data that identified genes as non-essential; however that result, while interesting to the Mycoplasma and Mycoplasma pneumoniae research community, it is not a story of broad interest like the story the authors wanted to tell.

At this juncture, I would suggest the EMBL/CRG group withdraw this manuscript and make a larger pool of mutants. They need to do what the Stanford Caulobacter group did. Importantly, they should do mutagenesis with either Tn5 or Tn4001, plate 100s of thousands of mutants, scrape them off the plate and serially passage them after growth 3 or 4 times. While this will enrich for fast growers, it will also dilute out any dead cells that contain disruptions in essential genes or genes

greatly reduce growth rate. Then, Illumina sequencing to identify transposon mutant locations would be done and the analysis methods the EMBL/CRG team has already established could be used. I would think this could all be done in a month is MiSeq instead of HiSeq sequencing was used.

NOTE from the Editor: Our additional correspondence with reviewer #2 regarding this point:

Our message to reviewer #2:

When you say that this could be done in a month do you mean the whole experiment (including repeating the mutagenesis screen) or do you refer only to the sequencing and data analysis? It would be important for us to clarify whether the whole experiment could be performed in a reasonable timeframe.

Response of reviewer #2:
Tn5 mutagenesis can be done from start to finish (meaning data analyzed) in just over 3 weeks, assuming MiSeq and not HiSeq is used, since HiSeq takes much longer (2 weeks as opposed to a day for MiSeq).

Additionally, removing some genes not expected to be disrupted from M. pneumoniae could validate some of the more surprising non-essential gene calls. Krishnakumar et al described an approach for doing this in their 2010 paper "Targeted chromosomal knockouts in Mycoplasma pneumoniae." This would give additional credibility to some surprising results. Without such a demonstration of gene removal, calling some genes non-essential based on transposon data is sometimes incredible. As an example the lead author on this paper, Maria Lluch-Senar demonstrated that M. genitalium was viable even without cell division protein FtsZ.

Once the analysis is redone with a statistically significant number of M. pneumoniae transposon bombardment mutants, this will be an amazing paper. The Stanford Caulobacter paper, because of the large genome size and genetic redundancy of the organism, likely underestimates the true fraction of bacterial genomes that is essential. In that genome there will be many duplicated or paralogous pairs of genes that encode essential functions but which are individually not essential. This will not be the case for M. pneumoniae, which is already near minimal, after a long evolution using gene loss to get to a very small, largely non-redundant genome designed for life in a very low stress niche.Because of that it will be extremely interesting to researchers who have no interest in mycoplasmas, but do care about bacterial evolution and the basic principals of cellular life.

I have a number of other less important criticisms of this paper. Most of those can be solved with simple editing.
Figure 1 labeling and explanation needs work. Affinity is mis-spelled, 22M reads needs some commas in the number, Q# & S# are not defined

Figure 2 legend what is TSC? There is a line at the bottom of the Aquiflez structure. What does that mean? No pdb found is cryptic.

From Author Contributions Section
This is a small error. The paper states: "MLS JDB and WHC assembled and analyzed the data and wrote the manuscript;" JDB is likely Javier Delgado. This should be fixed.

Legend Figure 1:

Haystack mutagenesis is incorrectly written as high stack mutagenesis.

Legend Figure 3.

There is a sentence stating: "The histogram represent the percentage of antisense ncRNAs that anticorrelate and correlate with their overlapping ORF along different time points of the growth curve." No growth curve is shown.

Supplementary Materials Page 3

The sentence "The 64 pools were ordered into II groups and genomic DNA of these II samples was performed with theIllustrabacteria genomic Kit (GE)." Presumably the missing words are "was isolated"

Supplementary Materials Page 4

This sentence needs to be revised: "Genomic DNAs were sheared to 100 bp DNA using a Covaris S2 device fragments."

This sentence also needs to be revised in order for it to make grammatical sense: "Paired-end Illumina libraries were created as described by Bentley et al. (Bentley et aI., 2008) and size selected between 200 and 400 bp."
Supplementary Materials Page 5

This statement begs explanation: "The uncertainty of the Maq-Blast insertion positioning method was 7 bp." I don't understand why the uncertainty and it also does not seem to equate to this statement in the main paper: "The resulting map consists of 34,825 unique mini-transposon insertions at a low resolution (-9 bp). "

Concerning the paragraph "Study of vias by GC content" there was an effort to determine if transposon insertions were localized based on AT%. First, vias should be bias I think. More importantly, in other studies using TN4001 in Mycoplasma genitalium and Mycoplasma pneumoniae (Glass et al. 2006, and Hutchison et al 1999), no correlation was observed between AT% and transposon insertion, but the insertions were certainly not random. Analysis of the HITS data does not get at this question. This only tells one the abundance of progeny of the 2976 different mutants after culture.

Supplementary Materials Page 7

This sentence mentions Table S8: "Cloning of domains ofMPN241, MPN 623, MPN 683 in pETM14 ccdb was done using Gibson assembly and the primers described in Table S8 (See Supplementary data)." No Table S8 was included for review.

Centrifugation conditions are inadequately described here: "After inductions and cell lysis by sonication, soluble and insoluble fractions were separated by centrifugation 15' at 15000 rpm." Give the value in g's.

Supplementary Materials Page 10

Centrifugation conditions reported as 15,000 rpm. Please tell us what centrifuge you used or how many g's.

Supplementary Materials Page 11

The table S5 is insufficiently labeled for readers to know what the column headers mean. This needs to be fixed.

Supplementary Materials Page 13
The sentence: "The different ORFs amplified by PCR and cloned by using Gibson Assembly Cloning Kit (New England Biolabs) into pMT85-clpB-taptag
SfiI/NotI digested vector." is missing the verb were between ORFs and amplified.

Reviewer #3:

Title: Defining a minimal cell: essential of small ORFs and ncRNAs in a genome-reduced bacterium
Authors: Maria Lluch-Senar et al.,

© European Molecular Biology Organization

This manuscript described the identification of essential genomic components for defining a minimal genome of Mycoplasma pneumonia based on the previously generated Tn-seq data with further computational and experimental analysis using newly annotated M. pneumonia genome. From their analyses, following issues have been analyzed and discussed.
Small ORFs (<100 aa), which account for 10% of all ORFs, has the largest group showing essentiality (57%) followed by conventional ORFs (53%).
Significant essentiality of ncRNA region
Domain essentiality rather than ORF by Pfam and InterPro
Antisense ncRNA that anticorrelate with their overlapping ORF along different time points of the growth curve showed more essentiality than those showing correlation.
Small ORF identification by MASS spectrometer
Protein complex analysis by molecular weight exclusion chromatography and Western blotting analysis using TAP-tagged 10 small ORFs.
Basically, I think this manuscript has a potential for the reader's interest, however, it has many rooms to be improved for publication.

The major concern is that this manuscript is not well organized. For the readability, the main text should be prepared minimal level to understand what they want to say, but I frequently check supplementary parts.
And I have some skepticism for Tn insertion mutagenesis. The original mutant library has 3000 mutants and is this number enough to saturate to cover this organism genome?

Minor issues are,
1) Page 3, L 17, "fitness" class is discussed without any explanation of this class. The definition of each of classes is defined in the page 6 of supplementary documents. And definitions of two classes of "essentiality" and "non-essentiality" were clearly defined in the early part in the section of "Essentiality score, gold standard sets". But the definition of "fitness" is explained in the last sentence of this section. I think this makes this manuscript not easy to read and gives the impression to the readers, at lease myself, as "not well organized". One solution might be that, clear definition of all three categories in the "Experimental Procedure", and put remarks, (see Experimental Procedure of Supplementary documents), in the main text.
2) Page 4, L 12, does usually the replication termination region show the essentiality. At least in E. coli, large deletion could be established in that region.
3) Page 4, L 13, can 5'-UTR region clearly be distinguishable between overlapped ncRNA?
4) Are the analyses of correlation or anti-correlation between overlapping ncRNA and ORFs possible to discussed about the essentiality of each of classes? I think it is not easy to distinguish the responsible cause of overlapped ncRNA and ORF. At least, it is not easy to understand how authors classified essentiality in overlapped region. More clear explanation may be required.
5) Page 5, L 13, for YlxR, the author referred the paper by Dammel and Noller (1995), however, I could not find it in that paper. How authors obtained the information of YlxR from the paper by Dammel? Or wrong paper was rreferred?
6) Page 5, L 18, authors made statement "suggesting that we discovered the near-to-complete M. pneumonia small proteome". But for me, it is too early to say so before performing currently available new approaches, such as ribosome profiling.
7) I have some ambiguity about the construction method of Tn insertion library. Can this method make sure to generate one Tn insertion per chromosome? Otherwise, multiple insertion mutations cannot be distinguished whose insertion is responsible for the growth fitness, or I have a wrong interpretation?
8) Page 7, L 13, table S8 is referred but I fail to find it.
9) In the same sentence, ccdb may be ccdB?
10) Page 10, L 5, authors analyzed 15 ul of samples by the gel, but I could not find the total volume to dissolve the sample.
11) Page 10, section ii) no fragmentation was performed? Whole protein was analyzed by MS? I cannot repeat the experiment by this information.
12) Page 11, the last sentence of section iii), what "B10 to E3" means?
13) Page 12, L 9, error during format exchange?
14) Page 14, both "ROC" and "AUC" should be shown their abbreviation, not just or "AUC".
15) Page 29 and 30, figure S6, this analysis may be the first issue to be discussed for the reliability of this mutant library, I think.

16) Generally speaking, quality of figures is not so good. For example,
a) Fig. 1C, it is hard to recognize Tn insertions.
b) I cannot understand correctly and easily what "the essentiality status of individual structural domains differs" means by Fig. 2
c) Fig. S1, what is the rule to align them?
d) Fig. S3, in graphs, distinguishable colors should be used. Blue and green are not easy to see.
e) Fig. S4, in the circle graph, blue and pale blue are the same as above.
f) For tables, legends describing each of columns are required.

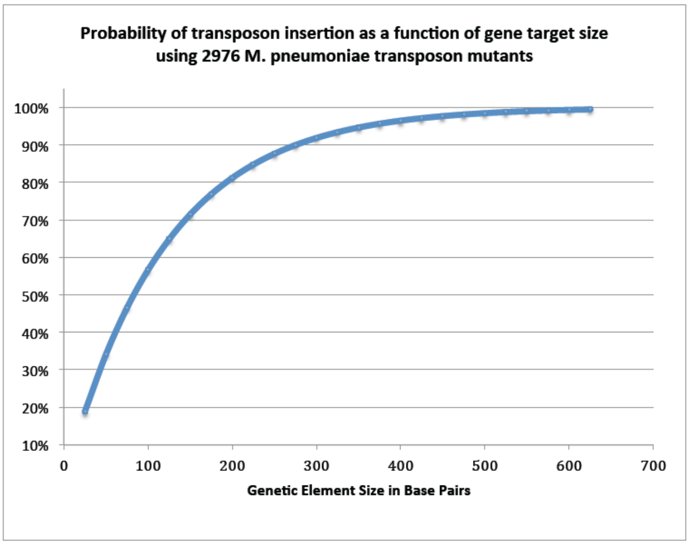

Chart 1: As shown in this chart, to have 99% confidence that a gene was hit among the 2976 M. pneumoniae mutants analyzed, the minimum target size would have to be about 550 base pairs.

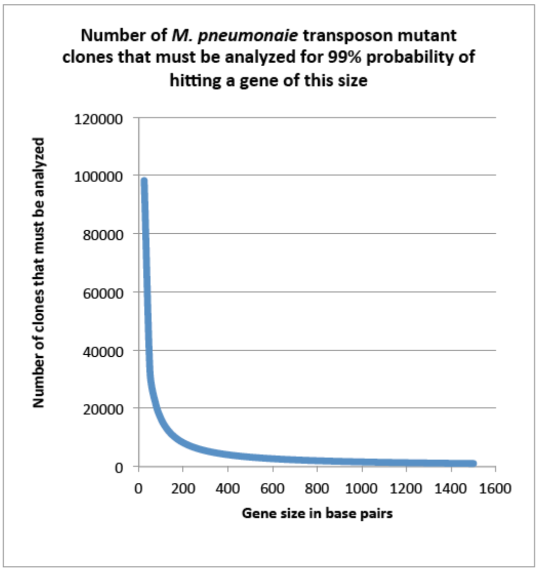

Chart 2: The conclusions about essential small ORFs and non-coding RNAs are not supported given they only analyzed a few less than 3000 mutants. They probably should have analyzed at least 100 thousand clones, and probably several times that amount.

1st Revision - authors' response 06 November 2014

REVIEWER 1:

General points:

© European Molecular Biology Organization

• *One of the questions around genome minimization is to determine the size of a minimal genome. The authors should have the means based on the present study to give at least a range of gene number/size of genome for a minimal genome starting with M. pneumoniae chassis.*
-Following the suggestion of reviewers 1 and 2 we have repeated the transposon analysis and now we have a much better coverage (4bp resolution for non-essential genes). We estimated that the percentage of essential and fitness regions are 33%, and 13%, respectively. This means we could, in principle and assuming no epistatic effects, remove 46% of the genome without dramatically affecting viability. The value 33%, resulting from the percentage of essential genomic elements, has been added into the abstract of the manuscript.

Genomic Regions	**%of genome**	**% of E**	**% of the essential genome**	**% of F**	**% of the fitness genome**
5'-UTRs	5	26	1,3	16,6	0,83
ncRNAs	26	5	1,3	12,1	3,146
Structural	10	0,9	0,09	6,25	0,625
ORFs	55	49	26,95	13,6	7,48
smORFs	5	53	2,65	11	0,55
Functional RNAs	1	82	0,82	6,1	0,061
		**% of whole genome that is Essential**	**33,11**	**% of whole genome that is Fitness**	**12,692**

• *For the translational machinery, a recent study published in PLoS Genetics by Grosjean et al suggested that the minimal protein machinery in Mollicutes would include 129 proteins/genes, a set slightly smaller than the translational gene set reported for M. pneumoniae in the same study. Are these 129 proteins/genes found as essential in the present study?*
Following the reviewer's suggestion, we compared our sets of essential genes to the one previously predicted byGrosjean et al..:
- In total 122 genes in *M. pneumoniae* were found to be orthologous to the *minimal protein machinery in Mollicutes* suggested by Grosjean et al (PMID: 24809820); among which, only two were nonessential (NE) in our experiment, the others were either essential (E, 112 (~92%)) or fitness (F, 8), as shown in Figure S10A. We identified 120 genes in *M. genitalium* genes orthologous to the Grosjean's list, and found that a much larger fraction (~18%) is nonessential (essentiality data obtained from (1)). See Table S11 for the data. We discuss this point on pages3, and 28-29, and on Figure S10.

• *M. genitalium and M. pneumoniae, although colonizing different mucosal surfaces or producing distinct human diseases, are very closely related bacteria. In a previous study by Glass et al (2006), it was indicated that "... some M. genitalium orthologs of nonessential M. pneumoniae genes are in fact essential". Does the present study confirm this suggestion? In fact, the results obtained in this 2006 paper could be compared with the results obtained here.*
We have performed the suggested analysis and included in the text (page 30 and Figure S10) as the referee suggests:
-444 pairs of one-to-one orthologous genes between *M. pneumoniae* and *M. genitalium* were extracted from firmNOG of eggNOG ver 4.0 (PMID: 24297252). Among which, about ~75% showed consistent essentiality statuses, i.e. essential or non-essential in the two mycoplasmas. The results did not change regardless to which group (essential (F==E; left stacked bar) or nonessential (F==NE; right stacked bar) the fitness genes identified in *M. pneumoniae* were assigned. See Table S11 for the data (Figure S10B). This information has been included in the section of ORF functional analysis of essentiality of the supplementary data. Also the figure S10 and Table S11 show the results of the analysis.
Minor comments on the main text and figures: p 2: I would remove « an accurate view of a minimal genome » from the abstract as it is not clear what it really means ;

-Changed by: "Our data highlights an unexpected hidden layer of smORFs with essential functions, as well as non-coding regions thus changing the focus when aiming to define the minimal essential genome".

p 3: - "...and NE (20 genes) strains": I believe that the authors mean "clones" rather than "strains"
-We agree with reviewer, the change has been included.
- "C. crescentus", the name of the genus needs to be provided as it was not cited before in the manuscript;
-We apologize for the mistake, the genus name has been provided. *p 4: - "showed autonomous folding"; this claim does not seem to be supported by the data* shown Fig. S2. Please clarify.
- We added this sentence to clarify: since they can be expressed in a soluble manner
- the origin of replication of bacterial chromosomes is usually abbreviated as oriC, and not as "ORI";

- The correct abbreviation has been included

p11: "Thermotoga" instead of "Termotoga"
-Changed

Figure 1: the essentiality genome map is too small, it is not possible to view the location of the insertions nor the gene names. It should be provided supplementary material. In Fig 1A, affinity is misspelled.

-The figure indicated the workflow of the project. We have edited the figure by changing the essentiality genome map for a circle with the essential regions marked in black. Also we supply the information of the annotation of *M. pneumoniae* genome as well as transposon insertion sites in a gbk file that we submit as supplementary data. This file can be launched in a genome browser to visualize properly all the transposon insertion sites. . Additionally we have provided genome position of the insertions of the different libraries in a supplementary table (Table S8).
Minor comments on Supplementary data: It would be useful for future studies to include a high resolution map of the transposon insertions and/or a table with all insertion positions
-We agree with the reviewer and a table with the insertion positions has been supplied in the supplementary data (Table S8). Additionally we provide the gbk files of the genome maps with the information of genomic elements and insertions for each one of the experiments.

• *Most of the figures in the supplementary materials should be enlarged for improving the manuscript readability;*
-We provide the pdfs of the figures in order to have them in better resolution and enlarged size.

• *p 7: concerning the expression of domains of putative modular proteins, was it necessary to avoid the UGA codons (coding for Trp in mycoplasmas)? This needs to be specified*
-Mutation of TGA (W in *M. pneumoniae* but Stop in *E. coli*) codons to TTG was required for protein expression of MPN683 and MPN623 in *E. coli*. We have included it into the text.

• *p 9: "Glycine" instead of "Glicine"*
-Changed

• *p 10 and elsewhere: misspelling, check: "M. pneumoniae" instead of "M. pneumoniae"*
-We have checked the misspelling.

• *p 12: a problem in the pdf production after «with 0.1% FA in 60 min at a flow of 0.3 »*
-The error has been corrected

• *p 20 : « In contrast, only 9 % of non-essential antisense cis- RNAs overlapped with essential ORFs ». How is it possible to conclude that a ncRNA is not essential when it is antisense from an essential CDS?*
The answer is that the beginning and end of essential genes could be non-essential (modularity) as has been shown by other groups. Thus, a ncRNA could overlap with these regions and be classified as non-essential. However, since we understand the concern of the reviewer, the percentage of essential ncRNAs has been calculated by considering only intergenic ncRNAs or ncRNAs overlapping with non-essential or fitness ORFs.

• p 20 and 33: « two of them [ncRNA] are conserved in other bacteria (ncMPN007 and ncMPN322; e-value < 0.005) ». It is not clear if these regions have been documented as being transcribed in other bacteria such as H. pylori. In addition, instead of provided an e-value from a Blast analysis, it would be more meaningful to provide a percentage of identity indicating the length of the aligned sequences.
-Homolog of a 56bp fragment of ncMPN007 can be found in H. pylori (E=0.005; seq-identity=63%) and is located in the intergenic region of two coding genes, HP0651 and HP0652. It it unclear whether the intergenic region in H. pylori is transcribed.

-Fragments of ncMPN322 are homologous at nucleotide level with some very remote species such as Vitis vinifera (E = 0.003; align-length=58; seq-identity=83%) and Protopolystoma xenopodis (E=0.011; align-length=71; seq-identity=79%). The matched regions are often at intergenic regions and not annotated as part of any coding gene.

-We have included the percentage of identity as the referee suggested.

REVIEWER 2:

Note that I have provided a Word file that includes some figures I have generated to better show the problem with this manuscript.
In an absolute sense, transposon bombardment only defines the non-essential regions of the genome. Even then, it is possible if the experiment is not set up properly to incorrectly label an essential gene as non-essential. For instance, a gene encoding a protein for which there are many copies in a cell, but for which only one copy is essential could be disrupted by a transposon and still result in cells that might survive 10 or more cell divisions. Thus, after the transposon reaction it is necessary to culture cells long enough that the entire pool of proteins initially present in transposon disrupted cell is exhausted.

To avoid this problem we repeated the experiments as suggested by the reviewer (see below) and we have done two serial passages and a total of 12 days of growth.

Similarly, as reported in both the Stanford Caulobacter paper and the Barcelona Mycoplasma manuscript being reviewed, disruption of some genes results in cells that have impaired growth phenotypes, called Fitness genes. To determine the essential regions of a genome one must make several assumptions about the transposon bombardment process:
• Transposon insertions are completely random
• The number of independent transposon mutants being analyzed is great enough that there is a high probability that all non-essential targets are hit.

I would suggest the EMBL/CRG group withdraw this manuscript and make a larger pool of mutants. They need to do what the Stanford Caulobacter group did. Importantly, they should do mutagenesis with either Tn5 or Tn4001, plate 100s of thousands of mutants, scrape them off the plate and serially passage them after growth 3 or 4 times. While this will enrich for fast growers, it will also dilute out any dead cells that contain disruptions in essential genes or genes greatly reduce growth rate. Then, Illumina sequencing to identify transposon mutant locations would be done and the analysis methods the EMBL/CRG team has already established could be used. I would think this could all be done in a month is MiSeq instead of HiSeq sequencing was used.
-We would like to thank the reviewer for the in depth analysis of our paper. -
We agree with the reviewer that a proper analysis of essentiality requires two critical factors: i) the randomness of the transposon insertions and ii) the pair base resolution of the study.

i) Randomness of the transposon insertions.
In reference to the use of Tn5 instead of Tn4001, in *M. pneumoniae* Tn5 is not the best tool for generating insertions (see below). The minitransposon derived from Tn4001 is the most currently used tool in transposon mutagenesis studies (2). In fact a recent paper by Green et al (2012; Mobile DNA) shows that "*Tn7 demonstrates markedly less insertion bias than either Mu or Tn5, **with both Mu and Tn5 biased toward sequences containing guanosine (G) and cytidine (C).** This preference of Mu and Tn5 yields less uniform spacing of insertions than for Tn7, in the adenosine (A) and thymidine (T) rich genome of C. glabrata (39% GC)*". Similarly in 2004 Ason et al showed a sequence bias for Tn5 in sites having a TTATA motif (JMB,335, 1213). All of this is confirmed in

a review in 2013 by Barquist et al (RNA biology, 10; 1161-1169). It seems Tn5 has a bias for certain sequences and a slight preference for A/T rich regions.
To find out if there is a bias in Tn4001 and to rule out a specific effect of antibiotics we have done two experiments one with a resistance to gentamycin and the other to tetracycline. After two serial passages and 12 days culture we found a correlation of 0.99 in the number of insertions per gene for both experiments indicating that the antibiotic used does not influence the insertion preferences. We did not detect any significant sequence A/T bias for 200 base regions when analyzing the essentiality of ORFs, ncMPNs and intergenic regions (Figure S5A in the current version of the manuscript). However, since Tn5 insertions have been shown to have a markedly stronger preference for an specific AT-rich 5-bp target sequence (3), we examined whether pMT85 and pMTnTetM438 have also a specific sequence bias. To do so and to have enough statistical power we analyzed the number of insertions per occurrence number in the genome for all possible quadruplets (256 combinations). We found that quadruplets having a high GC content have significantly less insertions per genome occurrence than those A/T rich (Figure S5B). Interestingly quadruplets starting with GC have overall more insertions per genome occurrence than others with similar G/C or A/T content ((Figure S5B). To ensure that this bias does not have an effect in our essentiality study for smORFs (5'UTR, ncRNAs and structural region are more A/T rich than coding genes) we determined the frequency of occurrence of the 256 quadruplets for smORFs and ORFs. As a control we did several random sets of ORFs. We find that the overall correlation of quadruplet frequencies between smORFs and conventional ORFs was very high (r=0.95) (Figure S5C), and similar to that found between random sets of ORFs. This shows that the transposon insertion bias is affecting equally both genomic categories.
All the study of transposon insertion bias has been included in the current version of the manuscript in the supplementary material (Study of bias by GC content section; page 14).

ii) Resolution
Regarding the haystack mutagenesis library, as the reviewer indicated the number of picked colonies was 2,976. However, we found 34,825 unique mini-transposon insertions. Since *M. pneumoniae* cells form aggregates, probably each colony could be derived from more of one clone. In fact, Glass et al. in 2006, showed that most of *M. genitalium* colonies were mixtures of two or more mutants and they referred to them and any DNA isolated from them as colonies rather than clones. We used the same terminology in our previous work to avoid misunderstandings. Furthermore, to experimentally corroborate the essentiality of the tested clones we had to filter several times and do serial passages to isolate single clones. Thus, the number of insertions corresponds to the number of clones and it is higher than the number of picked colonies. Looking at the figure that the reviewer sent, these 34,825 clones should be enough to identify at least 1 transposon insertion in a gene of 70 bp length with close to 99% probability. Thus, the coverage for small ORFs should be significant.

In any case we followed the instructions of reviewers 2 and 3 and did again the transposon library and repeated the deep sequencing analysis. Moreover we have used two different vectors (pMT85 and pMTnTetM438) harboring different antibiotic resistances (gentamycin and tetracycline) to eliminate possible specific effects of the chosen resistance. After independently transforming *M. pneumoniae* cells with the two minitransposon vectors (pMT85 and pMTnTetM438) 2 serial passages of cells and a total of 12 days growth were performed. This way we minimized insertions in essential genes that could be detected because of: i) the sensitivity of HITS technique. ii) the amount and/or half live of the protein could allow some rounds of cell division. iii) or cells that will not divide but survive for a long time. We confirmed that in each individual experiment, insertions in essential genes decrease whilst those numbers in non-essential remain approximately constant (Fig 1 this letter). Since the number of insertions per gene in each separated experiment was highly correlated (see Fig 2 this letter) we considered both experiments as biological replicates and merged the insertions.

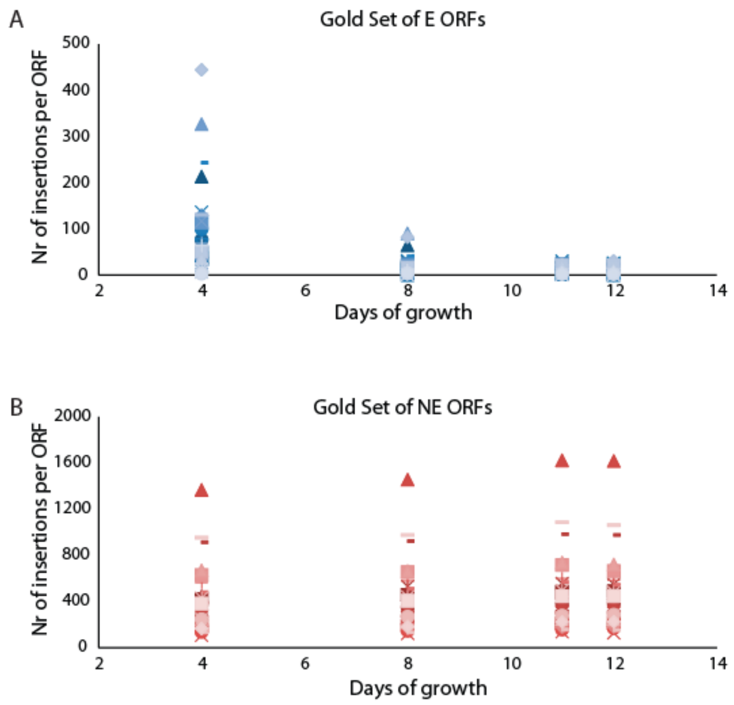

Figure 1. Densities of insertions in each gene of the different gold sets (essential E and not essential NE) after 2 cell passages corresponding to 12 days of growth.

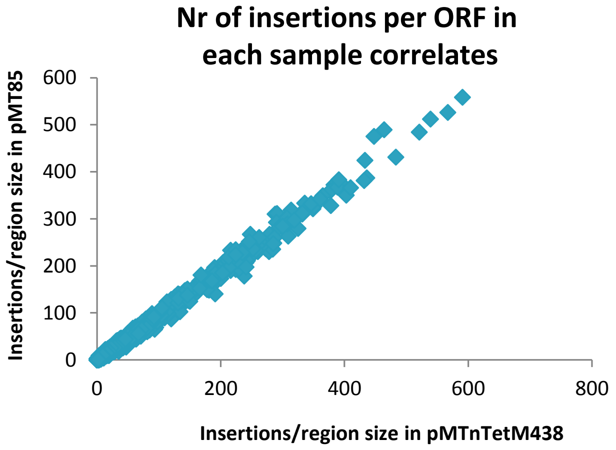

Figure 2. Correlation between densities of insertions per ORF in both transformations

After 12 days culture and two serial passages although as shown in Fig1 this letter we are close to a plateau, we still find a small number of insertions in the gold set. When looking at the number of reads per insertion we find a significant difference between the gold sets (mean of 7 in essential ORFs and 23 in non-essential). To decide on a threshold to consider an insertion noise or real, we did two analyses. The first one was a ROC curve (Threshold of 7 reads; AUC=0,76; TPR=0,63; FPR=0,22). In the second analysis we looked at the number of reads that will maximize the difference in density of insertion ratios between the essential and non-esssential gold sets (Figure 3 in this letter).

© European Molecular Biology Organization

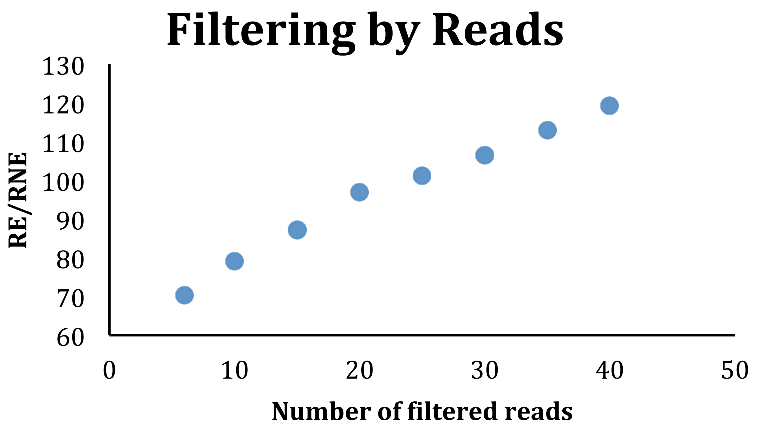

Figure 3. Filtering by reads in the two experiments. On the Y axis we show the ratio between insertion number in the essential and the non-essential gold sets. We chose 41 reads that is when the ratio seems to reach a plateau.

Based on these two analyses we set two thresholds, 7 reads per insertion (more relaxed) and 41 reads per insertion (close to the plateau of Fig. 3 this letter; more stringent). Both thresholds have their drawbacks. At 7 reads we found a significant number of functional RNAs (tRNAs, rRNAs and others) as fitness, while this number is very reduced at 40 reads. On the other hand three clones isolated by Stulke and co-workers are defined as fitness with 7 reads and essential with 40. Thus, we decided to leave both analysis in the suppl material tables for the readers and we used in our text the 40 reads cutoff but being aware that some fitness genes could be assigned to essential. This points to the problems of deep sequencing analysis of transposon libraries as discussed in (Barquist et al., 2013; 10; pp 1161).

Under the most stringent condition (minimum of 41 reads per insertion to consider an insertion valid) and considering the two transposon studies we have 69,994 unique mini-transposon insertions and a resolution of ~4 bp in non-essential genes. If we used the more relaxed 7 reads per then we have a total of 237,001 insertions). Looking at the graph done by the reviewer 2 with this number of insertions we can study the essentiality of a 70 bp chromosome region with a confidence higher than 99%.

The essentiality assignment and the different genomic regions have been done as in the previous version. The main messages of the previous version: smORFs are as essential as conventional ORFs, there are modular proteins in terms of essentiality and anticorrelated regulation of ncRNAs that overlap with essential ORFs, are the same. The only change is a decrease in the number of essential ncRNAs probably due to the greater coverage. So the main messages of the manuscript after addressing all the concerns of the different reviewers are not changing.

Additionally, removing some genes not expected to be disrupted from M. pneumoniae could validate some of the more surprising non-essential gene calls. Krishnakumar et al described an approach for doing this in their 2010 paper "Targeted chromosomal knockouts in Mycoplasma pneumoniae." This would give additional credibility to some surprising results. Without such a demonstration of gene removal, calling some genes non-essential based on transposon data is sometimes incredible. As an example the lead author on this paper, Maria Lluch-Senar demonstrated that M. genitalium was viable even without cell division protein FtsZ.

- The approach described by Krishnakumar et al to delete genes in *M. pneumoniae* showed a very low efficiency in the M129 strain (only one positive clone was described in the paper). To corroborate the essentiality determined in our study we isolated some clones (described in Table S2) from the pools of the Haystack library. Their purity has been assessed by Southern Blot and PCR. Additionally, in some cases (when antibody against the target protein was available) we performed Western Blot, and/or we did mass spectroscopy of some of the mutants (MPN223, MPN247, MPN248 (4); MPN241, MPN397, MPN420, MPN566) to find out if the protein corresponding to the gene inserted by the transposon was depleted. Regarding the FtsZ isolated by Lluch et al., it was shown that it is not essential when *M. pneumoniae* is grown on a plate, but it is in liquid culture. In our study it came out as fitness. Moreover, when looking at the 129 core genes suggested by Grosjean et al (PMID: 24809820), we found 122 ortologue genes in *M. pneumoniae*; among which, only two were nonessential (NE) in our experiment, the others were either essential (E, 112 (~92%))

© European Molecular Biology Organization

or fitness (F, 8), as shown in Figure S10A. As a comparison, we also identified 120 genes in *M. genitalium* genes that are orthologous to Grosjean's list, and found that a much larger fraction (~18%) is nonessential (essentiality data obtained from PMID:16407165). See Table S11 for the data.

I have a number of other less important criticisms of this paper. Most of those can be solved with simple editing. Figure 1 labeling and explanation needs work. Affinity is mis-spelled, 22M reads needs some commas in the number, Q# & S# are not defined
We have changed the figure and the explanation based on the new experiment and data analysis. We have explained better how we found the transposon insertion sites and we have simplified the figure by removing the genome map of essentiality.

Figure 2 legend what is TSC? There is a line at the bottom of the Aquiflez structure. What does that mean? No pdb found is cryptic.
TSC is translational start codon (we have included it in the text). We have eliminated it to avoid missunderstanding. The line indicates the start and the end of the sequence of the protein. We have included this information in the correspondent figure legend. Also the figure has been simplified by eliminating the probability graphs.
We apologize because the name of the bacteria was wrong. The orthologous protein was from *Methanocaldococcus jannaschii*, we have corrected it accordingly.

From Author Contributions Section: This is a small error. The paper states: "MLS JDB and WHC assembled and analyzed the data and wrote the manuscript;" JDB is likely Javier Delgado. This should be fixed.
It has been changed to JD.

Legend Figure 1: Haystack mutagenesis is incorrectly written as high stack mutagenesis.
-Changed

Legend Figure 3. There is a sentence stating: "The histogram represent the percentage of antisense ncRNAs that anticorrelate and correlate with their overlapping ORF along different time points of the growth curve." No growth curve is shown.
-A figure of *M. pneumoniae* growth curve and its legend have been included in the supplementary material (Figure S9) and it is referred in the legend of figure 3. *Supplementary Materials Page 3.*

The sentence "The 64 pools were ordered into II groups and genomic DNA of these II samples was performed with the Illustrabacteria genomic Kit (GE)." Presumably the missing words are "was isolated".
- We apologized for the mistake. In any case this does not apply to the new experiments.

Supplementary Materials Page 4. This sentence needs to be revised: "Genomic DNAs were sheared to 100 bp DNA using a Covaris S2 device fragments."
-The sentence has been changed by: “Genomic DNAs were sheared to 100 bp DNA fragments by using a Covaris S2 device”

This sentence also needs to be revised in order for it to make grammatical sense: "Paired-end Illumina libraries were created as described by Bentley et al. (Bentley et aI., 2008) and size selected between 200 and 400 bp."
-The sentence has been changed by: “After adapters ligation, generated fragments were size selected (between 200 and 400 bp)”.

Supplementary Materials Page 5. This statement begs explanation: "The uncertainty of the Maq-Blast insertion positioning method was 7 bp." I don't understand why the uncertainty and it also does not seem to equate to this statement in the main paper: "The resulting map consists of 34,825 unique mini-transposon insertions at a low resolution (-9 bp). "
-It has been previously described that in some cases the insertion of the transposon promotes the duplication of some bases of the insertion sites so some bases of the sequences read do not perfectly map to the genome region. We agree with the reviewer that it is not properly explained and it is misleading. The resolution by the mapping is 1 bp but in some very few cases the transposon

insertion can promote duplication in the insertion site and thus the insertion site assignment could have an uncertainty of 7 bp. We have changed it in the manuscript accordingly (page 12).

Concerning the paragraph "Study of vias by GC content" there was an effort to determine if transposon insertions were localized based on AT%. First, vias should be bias I think. More importantly, in other studies using TN4001 in Mycoplasma genitalium and Mycoplasma pneumoniae (Glass et al. 2006, and Hutchison et al 1999), no correlation was observed between AT% and transposon insertion, but the insertions were certainly not random. Analysis of the HITS data does not get at this question. This only tells one the abundance of progeny of the 2976 different mutants after culture.
Please see above in the section
i) Randomness of the transposon insertions

Supplementary Materials Page 7. This sentence mentions Table S8: "Cloning of domains ofMPN241, MPN623, MPN 683 in pETM14 ccdb was done using Gibson assembly and the primers described in Table S8 (See Supplementary data)." No Table S8 was included for review.
-We apologize for not including the table, it is now included in the current version as Table S9

Centrifugation conditions are inadequately described here: "After inductions and cell lysis by sonication, and insoluble fractions were separated by centrifugation 15' at 15000 rpm." Give the value in g's.
18,000 g by using a Beckman rotor JA20 is changed in all the instances of the manuscript

Supplementary Materials Page 10
Centrifugation conditions reported as 15,000 rpm. Please tell us what centrifuge you used or how many g's.
18,000 g by using a Beckman rotor JA20

Supplementary Materials Page 11
The table S5 is insufficiently labeled for readers to know what the column headers mean. This needs to be fixed.
We have described more in detail the table legend

Supplementary Materials Page 13
The sentence: "The different ORFs amplified by PCR and cloned by using Gibson Assembly Cloning Kit (New England Biolabs) into pMT85-clpB-taptag
SfiI/NotI digested vector." is missing the verb were between ORFs and amplified.
We apologize for the mistake. The verb has been included in the text..

REVIEWER 3:
The major concern is that this manuscript is not well organized. For the readability, the main text should be prepared minimal level to understand what they want to say, but I frequently check supplementary parts.
And I have some skepticism for Tn insertion mutagenesis. The original mutant library has 3000 mutants and is this number enough to saturate to cover this organism genome?
-We understand the major concern of the referee and we try to address his comment about Tn mutagenesis saturation.
Regarding the haystack mutagenesis library, as the reviewer indicated the number of picked colonies was 2,976. However, we found 34,825 unique mini-transposon insertions. Since *M. pneumoniae* cells form aggregates, probably each colony could be derived from more of one clone. In fact, Glass et al. in 2006, showed that most of *M. genitalium* colonies were mixtures of two or more mutants and they referred to them and any DNA isolated from them as colonies rather than clones. We used the same terminology in our previous work to avoid misunderstandings. Furthermore, to experimentally corroborate the essentiality of the tested clones we had to filter several times and do serial passages to isolate single clones. Thus, the number of insertions corresponds to the number of clones and it is higher than the number of picked colonies. Looking at the figure that the reviewer sent, these 34,825 clones should be enough to identify at least 1 transposon insertion in a gene of 70 bp length with close to 99% probability. Thus, the coverage for small ORFs should be significant.

© European Molecular Biology Organization

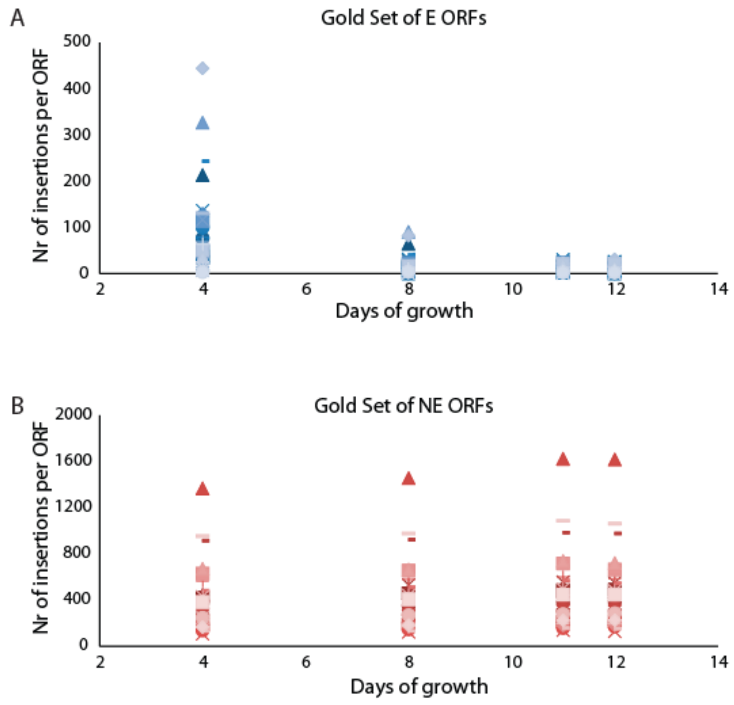

In any case we followed the instructions of reviewers 2 and 3 and did again the transposon library and repeated the deep sequencing analysis. Moreover we have used two different vectors (pMT85 and pMTnTetM438) harboring different antibiotic resistances (gentamycin and tetracycline) to eliminate possible specific effects of the chosen resistance. After independently transforming *M. pneumoniae* cells with the two minitransposon vectors (pMT85 and pMTnTetM438) 2 serial passages of cells and a total of 12 days growth were performed. This way we minimized insertions in essential genes that could be detected because of: i) the sensitivity of HITS technique. ii) the amount and/or half live of the protein could allow some rounds of cell division. iii) or cells that will not divide but survive for a long time. We confirmed that in each individual experiment, insertions in essential genes decrease whilst those numbers in non-essential remain approximately constant (Fig 1 this letter). Since the number of insertions per gene in each separated experiment was highly correlated (see Fig 2 this letter) we considered both experiments as biological replicates and merged the insertions.

Figure 1. Densities of insertions in each gene of the different gold sets (essential E and not essential NE) after 2 cell passages corresponding to 12 days of growth.

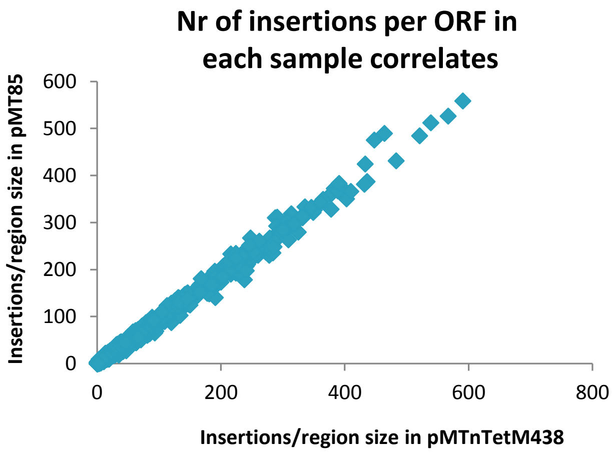

Figure 2. Correlation between densities of insertions per ORF in both transformations

After 12 days culture and two serial passages although as shown in Fig1 this letter we are close to a plateau, we still find a small number of insertions in the gold set. When looking at the number of reads per insertion we find a significant difference between the gold sets (mean of 7 in essential ORFs and 23 in non-essential). To decide on a threshold to consider an insertion noise or real, we

did two analyses. The first one was a ROC curve (Threshold of 7 reads; AUC=0,76; TPR=0,63; FPR=0,22). In the second analysis we looked at the number of reads that will maximize the difference in density of insertion ratios between the essential and non-esssential gold sets (Figure 3 in this letter).

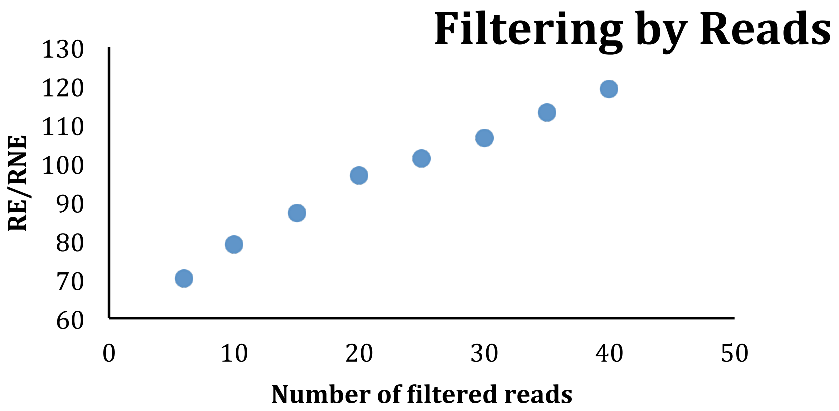

Figure 3. Filtering by reads in the two experiments. On the Y axis we show the ratio between insertion number in the essential and the non-essential gold sets. We chose 41 reads that is when the ratio seems to reach a plateau.

Based on these two analyses we set two thresholds, 7 reads per insertion (more relaxed) and 41 reads per insertion (close to the plateau of Fig. 3 this letter; more stringent). Both thresholds have their drawbacks. At 7 reads we found a significant number of functional RNAs (tRNAs, rRNAs and others) as fitness, while this number is very reduced at 40 reads. On the other hand three clones isolated by Stulke and co-workers are defined as fitness with 7 reads and essential with 40. Thus, we decided to leave both analysis in the suppl material tables for the readers and we used in our text the 40 reads cutoff but being aware that some fitness genes could be assigned to essential. This points to the problems of deep sequencing analysis of transposon libraries as discussed in (Barquist et al., 2013; 10; pp 1161).

Under the most stringent condition (minimum of 41 reads per insertion to consider an insertion valid) and considering the two transposon studies we have 69,994 unique mini-transposon insertions and a resolution of ~4 bp in non-essential genes. If we used the more relaxed 7 reads per then we have a total of 237,001 insertions). Looking at the graph done by the reviewer 2 with this number of insertions we can study the essentiality of a 70 bp chromosome region with a confidence higher than 99%.

The essentiality assignment and the different genomic regions have been done as in the previous version. The main messages of the previous version: smORFs are as essential as conventional ORFs, there are modular proteins in terms of essentiality and anticorrelated regulation of ncRNAs that overlap with essential ORFs, are the same. The only change is a decrease in the number of essential ncRNAs probably due to the greater coverage. So the main messages of the manuscript after addressing all the concerns of the different reviewers are not changing.

The definition of each of classes is defined in the page 6 of supplementary documents. And definitions of two classes of "essentiality" and "non-essentiality" were clearly defined in the early part in the section of "Essentiality score, gold standard sets". But the definition of "fitness" is explained in the last sentence of this section. I think this makes this manuscript not easy to read and gives the impression to the readers, at lease myself, as "not well organized". One solution might be that, clear definition of all three categories in the "Experimental Procedure", and put remarks, (see Experimental Procedure of Supplementary documents), in the main text.
-We have followed the recommendation of the reviewer and we have included the definition of the three categories in the "Experimental Procedure" section and added the suggested remarks.

2) Page 4, L 12, does usually the replication termination region show the essentiality. At least in E. coli, large deletion could be established in that region.
-In our new transposon library analysis by having higher resolution we find that indeed the Ori is essential but not the Ter.

3) Page 4, L 13, can 5'-UTR region clearly be distinguishable between overlapped ncRNA?
-When a ncRNA or an ORF is overlapping with the 5'-UTR this regions have been discarded in the essentiality assignment.

4) Are the analyses of correlation or anti-correlation between overlapping ncRNA and ORFs possible to discussed about the essentiality of each of classes? I think it is not easy to distinguish the responsible cause of overlapped ncRNA and ORF. At least, it is not easy to understand how authors classified essentiality in overlapped region. More clear explanation may be required.
A non-coding RNA can be non-essential and overlap partially with the 3' or 5'of the correspondent ORF. After repeating the transposon library, the resolution is 1 insertion each 4 bp. This level of resolution allows assessing the essentiality for the defined region of the ncRNA independently of the essentiality of the overlapping ORF if the overlapping region does not comprise all the ORF. For this reason we removed the overlapping region of these ncRNAs that are overlapping and we recalculate the essentiality of the remaining region for the ncRNA longer than 100 bp. However since we understand the concern of the reviewer, the percentage of essential ncRNAs has been calculated by considering only intergenic ncRNAs or ncRNAs overlapping with non-essential or fitness ORFs.
-The correlation and anticorrelation of gene expressions are assessed first independently of genes essentiality and the main conclusion is that ncRNAs and overlapping ORFS show more anticorrelation along growth curve in E than in NE ORFs suggesting putative regulatory role.

5) Page 5, L 13, for YlxR, the author referred the paper by Dammel and Noller (1995), however, I could not find it in that paper. How authors obtained the information of YlxR from the paper by Dammel? Or wrong paper was rreferred?
We apologize for the mistake it was an error with endnote program. We have put the correct citation.

6) Page 5, L 18, authors made statement "suggesting that we discovered the near-to-complete M. pneumonia small proteome". But for me, it is too early to say so before performing currently available new approaches, such as ribosome profiling.
We have re-written the sentence by: "suggesting that we are closer to define the complete *M. pneumoniae* small proteome"

7) I have some ambiguity about the construction method of Tn insertion library. Can this method make sure to generate one Tn insertion per chromosome? Otherwise, multiple insertion mutations cannot be distinguished whose insertion is responsible for the growth fitness, or I have a wrong interpretation?
In the transposon analysis of *M. genitalium* where they the same transposon, isolated individual clones (52 colonies) and sequenced them they found only one insertion per clone (2). This does not exclude the possibility that in some very few clones two transposons could be inserted but the number will be minimal. Since we base our classification not on single insertions but on overall density we think this will be negligible.

8) Page 7, L 13, table S8 is referred but I fail to find it.
We have included the table with the primers information as Table S9.

9) In the same sentence, ccdb may be ccdB?
We apologize for the mistake, we have changed accordingly.

10) Page 10, L 5, authors analyzed 15 ul of samples by the gel, but I could not find the total volume to dissolve the sample.
We have specified the final volume of 200 ul in the text as the reviewer suggested.

11) Page 10, section ii) no fragmentation was performed? Whole protein was analyzed by MS? I cannot repeat the experiment by this information.
We apologized the samples were digested with Trypsin and then injected in the mass spec (see **Proteomics analysis in the suppl material section)**.

12) Page 11, the last sentence of section iii), what "B10 to E3" means?

-We agree with the referee that the sentence was not correct. We have changed the sentence to avoid misunderstanding: “Fractions of molecular weight exclusion chromatography corresponding to elution volumes 7.5 ml to 25.5 ml (samples named B10 to E3), were analyzed by MS and Western Blot”.

13) Page 12, L 9, error during format exchange?
-We could not identify the mistake in the current version it was probably a problem during format change.

14) Page 14, both "ROC" and "AUC" should be shown their abbreviation, not just or "AUC".
We have included the definition for ROC (Receiving Operating Characteristic curve) the first instance that it appears in the text.

15) Page 29 and 30, figure S6, this analysis may be the first issue to be discussed for the reliability of this mutant library, I think.
The analysis has changed so we have repeated the vias by GC content and there is not insertion bias by the GC content by using miniTN4001 vectors (See Figure S5 of the curresnt version of the manuscript)..

16) Generally speaking, quality of figures is not so good. For example,
-We have edited all the figures to improve the resolution and we are attaching the pdfs.
a) Fig. 1C, it is hard to recognize Tn insertions.
-This figure has been edited. We are providing a gbk file that includes all the genome information including also the transposon insertion sites and it can be launched in a genome browser that allow the visualization at high resolution.
b) I cannot understand correctly and easily what "the essentiality status of individual structural domains differs" means by Fig. 2
-It means that individual pfam domains show different essentiality and they also correspond to different protein domains.
c) Fig. S1, what is the rule to align them?
-We apologize; we do not understand the question…
d) Fig. S3, in graphs, distinguishable colors should be used. Blue and green are not easy to see.
This figure has been removed because we think that is not relevant in the last version of the manuscript.
e) Fig. S4, in the circle graph, blue and pale blue are the same as above.
They are the same color because they are the same category.
f) For tables, legends describing each of columns are required.
We have described all the columns of tables in legends of supplementary tables.

1. Glass, J.I., Assad-Garcia, N., Alperovich, N., Yooseph, S., Lewis, M.R., Maruf, M., Hutchison, C.A., 3rd, Smith, H.O. and Venter, J.C. (2006) Essential genes of a minimal bacterium. *Proceedings of the National Academy of Sciences of the United States of America*, **103**, 425-430.
2. Lluch-Senar, M., Vallmitjana, M., Querol, E. and Pinol, J. (2007) A new promoterless reporter vector reveals antisense transcription in Mycoplasma genitalium. *Microbiology*, **153**, 2743-2752.
3. Kumar, A., Seringhaus, M., Biery, M.C., Sarnovsky, R.J., Umansky, L., Piccirillo, S., Heidtman, M., Cheung, K.H., Dobry, C.J., Gerstein, M.B. *et al.* (2004) Large-scale mutagenesis of the yeast genome using a Tn7-derived multipurpose transposon. *Genome research*, **14**, 1975-1986.
4. van Noort, V., Seebacher, J., Bader, S., Mohammed, S., Vonkova, I., Betts, M.J., Kuhner, S., Kumar, R., Maier, T., O'Flaherty, M. *et al.* (2012) Cross-talk between phosphorylation and lysine acetylation in a genome-reduced bacterium. *Molecular systems biology*, **8**, 571.

2nd Editorial Decision 04 December 2014

Thank you again for submitting your work to Molecular Systems Biology. We have now heard back from the two referees who agreed to evaluate your manuscript. As you will see from the reports below, the referees are satisfied with the modifications made and they think that the study is now suitable for publication.

Before formally accepting the manuscript we would like to ask you to deposit the MS data in one of the major public databases and to provide the dataset identifier in the "Data Availability" section of your manuscript.

Reviewer #2:

Maria Lluch-Senar and her colleagues have done an excellent job in their revision of this manuscript. They have thoroughly addressed each of the comments and suggestion offered by the three referees. I in particular was well satisfied with the way the text has been clarified and improved and with the new experiments that were performed. Over the last 80 years there have been some 1800 scientific publications that address the idea of a minimal bacterial cell; although none have ever actually had a real minimal cell to work with. Clearly this is an important topic to many biologists. Dr. Lluch-Senar's current paper, in my view, while not actually describing an extant minimal cell, comes closer to defining what would be part of such a cell than any of the previous papers on this topic that I am familiar with. I urge Molecular Systems Biology to publish this. It will be widely read and discussed by serious scientists who think about the origins of cellular life, systems biology, and bacterial physiology. I say bravo to Dr., Lluch-Senar, Dr. Serrano,and all their colleagues.

Reviewer #3:

This manuscript has been greatly improved. The authors did construction of the transposon mutant library again and analyzed. This makes the current analysis much more reliable. And I think their analyses have been performed properly with good statistical reliability.
And this analysis may have benefits for readers of the journal.
I think this manuscript is worth to be published.

2nd Revision - authors' response 15 December 2014

We have submitted the raw and analyzed data of MS in the Pride database following your indications and we have included the accession numbers in the current version of the manuscript.
Also, the manuscript has been submitted in the webpage with the supplementary tables in separated excel files and the bibliography formatted as was asked.
Once more, thank you very much for accepting our manuscript.

© European Molecular Biology Organization
